# Allogeneic mesenchymal stem cell therapy with laromestrocel in mild Alzheimer’s disease: a randomized controlled phase 2a trial

**DOI:** 10.1038/s41591-025-03559-0

**Published:** 2025-03-10

**Authors:** Brian G. Rash, Kevin N. Ramdas, Nataliya Agafonova, Eric Naioti, Lisa McClain-Moss, Zarin Zainul, Brittany Varnado, Kevin Peterson, Michael Brown, Thiago Leal, Steven Kopcho, Raul Carballosa, Paayal Patel, Mark Brody, Brad Herskowitz, Ana Fuquay, Savannah Rodriguez, Alan F. Jacobson, Ramon Leon, Michael Pfeffer, Julie B. Schwartzbard, Jeffrey Botbyl, Anthony A. Oliva, Joshua M. Hare

**Affiliations:** 1 Longeveron, Miami, FL USA; 2 First Excellent Research, Miami, FL USA; 3 Brain Matters Research, Delray Beach, FL USA; 4 Brainstorm Research, Miami, FL USA; 5 Allied Clinical Trials, Miami, FL USA; 6 Fusion Medical & Research Clinic, Miami, FL USA; 7 Imic, Palmetto Bay, FL USA; 8 Visionary Investigators Network, Aventura, FL USA; 9 Interdisciplinary Stem Cell Institute, Miller School of Medicine, University of Miami, Miami, FL USA

**Keywords:** Stem-cell therapies, Alzheimer's disease, Alzheimer's disease

## Abstract

Alzheimer’s disease (AD) is characterized by progressive cognitive decline, severe brain atrophy and neuroinflammation. We conducted a randomized, double-blind, placebo-controlled, parallel-group phase 2a clinical trial that tested the safety and efficacy of laromestrocel, a bone-marrow-derived, allogeneic mesenchymal stem-cell therapy, in slowing AD clinical progression, atrophy and neuroinflammation. Participants across ten centers in the United States were randomly assigned 1:1:1:1 to four infusion groups: group 1 (placebo; four monthly infusions, *n* = 12); group 2 (25 million cells, one infusion followed by three monthly infusions of placebo, *n* = 13); group 3 (25 million cells; four monthly doses, *n* = 13); and group 4 (100 million cells; four monthly doses, *n* = 11). The study met its primary end point of safety; the rate of treatment-emergent serious adverse events within 4 weeks of any infusion was similar in all four groups: group 1, 0% (95% CI 0–26.5%); group 2, 7.7% (95% CI 0.2–36%); group 3, 7.7% (95% CI 0.2–36%) and group 4, 9.1% (95% CI 0.2–41.3%). Additionally, there were no reported infusion-related reactions, hypersensitivities or amyloid-related imaging abnormalities. Laromestrocel improved clinical assessments at 39 weeks compared to placebo, as measured by a composite AD score (secondary end point was met: group 2 versus placebo change: 0.38; 95% CI −0.06–0.82), Montreal cognitive assessment and the Alzheimer’s Disease Cooperative Study Activities of Daily Living. At 39 weeks, Laromestrocel slowed the decline of whole brain volume compared to placebo (*n* = 10) by 48.4% for all treatment groups combined (groups 2–4: *P* = 0.005; *n* = 32) and left hippocampal volume by 61.9% (groups 2–4, *P* = 0.021; *n* = 32), and reduced neuroinflammation as measured by diffusion tensor imaging. The change in bilateral hippocampal atrophy correlated with the change in mini-mental state exam scores (*R* = 0.41, *P* = 0.0075) in all study patients (*N* = 42). Collectively these results support safety of single and multiple doses of laromestrocel treatment for mild AD and provide indications of efficacy in combating decline of brain volume and potentially cognitive function. Larger-scale clinical trials of laromestrocel in AD are warranted. ClinicalTrials.gov registration: NCT05233774.

## Abstract (web summary)

Single and multiple intravenous infusions with laromestrocel, an allogeneic cell therapy, is safe in patients with mild AD and may reduce the impact of cognitive decline and brain atrophy within 9 months of treatment.

## Main

AD is a devastating and progressive neurocognitive disorder representing the primary cause of dementia^1–5^. Until recently, AD treatments lacked disease-modifying effects and an AD diagnosis led to inevitable and disabling cognitive decline in all affected individuals^6^. Attempts to modify the disease have focused largely on the amyloid and neurofibrillary tangle hypotheses of disease pathogenesis^7^. After many failures of monoclonal antibodies attempting to remove amyloid proteins from the brain, three drugs were approved by the US Food and Drug Administration (FDA) between 2021 and 2024^8^.

In a phase 3 trial of lecanemab, randomization to active treatment resulted in a 27% improvement in Clinical Dementia Rating scale sum of boxes (CDR-SB) at 18 months^9–12^. Despite this therapeutic advance, lecanemab is associated with a 21.5% risk of amyloid-related imaging abnormalities (ARIAs)^13,14^ and the possibility of continued progression of brain atrophy^15,16^. Additionally, lecanemab resulted in a 26.4% rate of infusion-related reactions that can be devastating for a patient. Aducanumab carried a 35% risk of ARIA-edema (ARIA-E) and 19% risk of ARIA-hemorrhage (ARIA-H)^17^. Further, a third anti-amyloid drug, donanemab, carries a 36.8% risk of ARIAs (ARIA-E or ARIA-H), with drug-induced inflammation and brain bleeding reported^18^. Accordingly, it is imperative to continue to develop novel therapeutic strategies.

Nonamyloid and non-tau contributions to AD include cerebrovascular degradation and a neuroinflammatory component contributing to neuronal death and brain atrophy^19,20^. The neurovascular unit, composed of endothelial cells lining cerebral vessels in tight association with pericytes and astrocytes, regulates cerebral blood flow and the exchange of nutrients and waste products generated by the substantial energetic demands of active neural networks, and is the central component of the blood–brain barrier (BBB)^20,21^. Dysregulation of this system is often observed along with hallmark neuroinflammation in AD, but the precise relationship of these features with amyloid and tau pathology remains a matter of debate^22–24^.

Cell-based therapy with mesenchymal stem cells (MSCs) is a particularly attractive treatment candidate as it encompasses provascular, anti-inflammatory, immunomodulatory and tissue repair mechanisms of action, with findings validated in a murine AD model^25,26^. For AD, these effects could represent a neuroprotective dimension to treatment that mitigates some of the more destructive aspects of the disease and augments anti-amyloid/tau regimens. We have previously shown in a phase I study that laromestrocel (Lomecel-B) is safe in patients with mild AD and does not cause ARIAs, including in ApoE4 homozygous carriers^27^. Laromestrocel is immunoprivileged and is administered as an allogeneic cell therapy, due in part to undetectable expression of major histocompatibility complex (MHC)-II molecules, low-level MHC-I expression, and immunomodulatory properties^25,26,28,29^.

To advance our understanding of the potential therapeutic use of laromestrocel in AD, we conducted a 49-patient randomized, double-blind, placebo-controlled phase 2a study testing three different dosing regimens of laromestrocel. Our objectives were to test the hypothesis that intravenous administration of laromestrocel was safe, could ameliorate both the clinical disease progression of AD and its associated brain atrophy, and to gather mechanistic insight on potential roles in influencing disease trajectory.

## Results

### Patient disposition

**Fig. 1:**
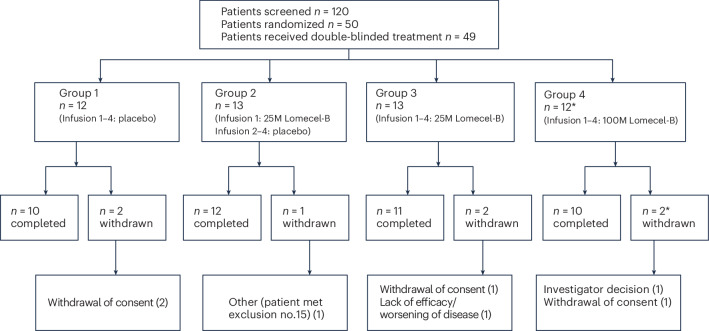
Consort diagram depicting study design. Full analysis set (FAS): *N* = 120; per-protocol population (PP): *N* = 41; ITT population: *N* = 49; mITT population: *N* = 48.

A total of 120 patients with a clinical diagnosis of mild AD were screened and 50 were enrolled. The first patient was enrolled on 29 December 2021 and the last on 21 November 2022, with the last patient study completion on 21 August 2023. All eligible patients were randomized 1:1:1:1 in block sizes of four, with each site receiving separate blocks. Randomization was additionally stratified by sex. Forty-nine randomized participants received double-blind treatment and were analyzed for the primary end point, safety, as part of the intention-to-treat (ITT) population (Fig. 1). One patient randomized to group 4 was not treated based on the investigator’s decision. The remaining 48 patients completed treatment with at least one post-baseline efficacy assessment and were analyzed for the secondary and exploratory end points as part of the modified ITT (mITT) population. Laromestrocel was administered through intravenous infusions of approximately 40 min duration with no pre-medication required. The treatment groups in the mITT population included: group 1, placebo infusions once per month for 4 months (*n* = 12); group 2, 25 million cells at day 0 followed by three monthly infusions of placebo (25 M × 1; *n* = 12); group 3, 25 million cells infused monthly for 4 months (25 M × 4; *n* = 13); and group 4: 100 million cells infused monthly for 4 months (100 M × 4; *n* = 11) (Fig. 1).

Baseline demographics and disease characteristics for the ITT population are presented in Table 1. Overall, the mean ± s.d. age was 74.1 ± 6.65 years (*n* = 49), with a lower proportion of male (44.9%) versus female (55.1%) patients. The majority of patients were white (95.9%) with a higher proportion of Hispanic/Latino ethnicity (75.5%). Medication for symptomatic AD at baseline was taken by 63.3% of patients. Baseline volumetric magnetic resonance imaging (MRI) values are shown in Supplementary Table 1 and inclusion/exclusion criteria are shown in Supplementary Table 2. All study patients, caregivers and all personnel involved in the conduct and interpretation of the study, including investigators, site personnel and sponsor staff, were blinded to treatment group.

**Table 1:** Demographics and baseline characteristics (ITT population)

Parameter	Group 1 (placebo) (*N* = 12)	Group 2 (25 M × 1) (*N* = 13)	Group 3 (25 M × 4) (*N* = 13)	Group 4 (100 M × 4) (*N* = 11)	All patients (*N* = 49)	*P* value
Age, years, mean (s.d.)	76.7 (4.40)	74.9 (7.51)	70.5 (7.14)	74.5 (6.07)	74.1 (6.65)	0.116
Sex						0.875
Male, *n* (%)	5 (41.7)	5 (38.5)	7 (53.8)	5 (45.5)	22 (44.9)	
Female, *n* (%)	7 (58.3)	8 (61.5)	6 (46.2)	6 (54.5)	27 (55.1)	
Race						0.066
Black/African American, *n* (%)	0	0	0	2 (18.2)	2 (4.1)	
White, *n* (%)	12 (100.0)	13 (100.0)	13 (100.0)	9 (81.8)	47 (95.9)	
Ethnicity						0.697
Hispanic/Latino, *n* (%)	9 (75.0)	11 (84.6)	10 (76.9)	7 (63.6)	37 (75.5)	
Not Hispanic/Latino, *n* (%)	3 (25.0)	2 (15.4)	3 (23.1)	4 (36.4)	12 (24.5)	
ApoE4 status						0.697
Non-carrier, *n* (%)	10 (83.3)	8 (61.5)	7 (53.8)	10 (90.9)	35 (71.4)	
Heterozygous, *n* (%)	2 (16.7)	5 (41.7)	5 (38.5)	1 (8.3)	13 (26.5)	
Homozygous, *n* (%)	0	0	1 (7.7)	0	1 (2.0)	
AD treatment, *n* (%)	7 (58.3)	9 (69.2)	8 (61.5)	7 (63.6)	31 (63.3)	0.952
Donepezil, *n* (%)	5 (41.7)	6 (46.2)	7 (53.8)	5 (45.5)	23 (46.9)	
Memantine, *n* (%)	3 (25.0)	4 (30.8)	5 (38.5)	3 (27.3)	15 (30.6)	
Donepezil/memantine, *n* (%)	0	1 (7.7)	0	0	1 (2.0)	
Galantamine, *n* (%)	0	0	1 (7.7)	0	1 (2.0)	
Height (cm), mean (s.d.)	165.0 (14.2)	170.2 (8.6)	166.5 (10.1)	167.1 (9.1)	167.3 (10.6)	0.665
Weight (kg), mean (s.d.)	79.5 (15.5)	78.6 (16.6)	84.1 (14.9)	77.8 (14.8)	80.1 (15.2)	0.741
BMI (kg m^−2^), mean (s.d.)	30.4 (6.63)	27.1 (5.35)	30.4 (5.39)	27.8 (4.06)	28.9 (5.49)	0.707
MoCA total score, mean (s.d.)	19.0 (5.83)	16.0 (4.51)	19.2 (4.18)	18.2 (4.58)	18.1 (4.83)	0.772
MMSE-2 total score, mean (s.d.)	20.9 (2.27)	20.8 (2.23)	21.9 (2.06)	21.2 (1.60)	21.2 (2.05)	0.533
ADAS-Cog-13 total score, mean (s.d.)	31.30 (11.82)	32.92 (8.87)	27.87 (8.91)	23.58 (4.13)	29.09 (9.36)	0.071
CDR-SB total score, mean (s.d.)	3.75 (1.37)	5.19 (1.52)	3.73 (1.56)	4.23 (1.72)	4.23 (1.62)	0.071
ADCS-ADL total score, mean (s.d.)	59.1 (9.10)	55.8 (9.24)	61.9 (13.43)	63.1 (8.95)	59.9 (10.49)	0.322

BMI, body mass index. Group 1, placebo (dose × 4). Group 2, laromestrocel (25 M × 1). Group 3, laromestrocel (25 M × 4). Group 4, laromestrocel (100 M × 4). Baseline cognitive and functional tests represent the scores of the last test before the first dose. The ITT population was *n* = 49; after one withdrawal from group 2, the mITT was *n* = 48.

### Primary outcome

The study met its primary end point (Table 2). The primary end point was assessed by measuring the proportion of patients who experienced at least one treatment-emergent (TE) serious adverse event (SAE) within 4 weeks of any infusion. Additionally, safety was evaluated by the incidence of all adverse events (AEs) and SAEs throughout the study, clinical laboratory assessments, physical examinations and ARIAs (ARIA-H or ARIA-E) or clinically asymptomatic microhemorrhages as assessed by MRI.

**Table 2:** Safety summary (ITT)

Adverse events	Statistic	Group 1 (placebo; *N* = 12)	Group 2 (25 M × 1; *N* = 13)	Group 3 (25 M × 4; *N* = 13)	Group 4 (100 M × 4; *N* = 11)	All patients (*N* = 49)
Total number of TE-AEs	*n*	18	21	20	24	83
Total number of TE-SAEs	*n*	0	1	2	3	6
Total number of grade 3 or grade 4 TE-AEs	*n*	1	1	2	3	7
Total number of related TE-AEs	*n*	0	1	2	0	3
Total number of related TE-SAEs	*n*	0	0	0	0	0
Total number of TE-AEs Leading to study discontinuation	*n*	0	0	0	0	0
Total number of TE-AEs leading to treatment discontinuation	*n*	0	0	0	0	0
Total number of TE-AEs leading to treatment interruption	*n*	3	0	1	0	4
Total number of TE-SAEs with outcome of death	*n*	0	0	0	0	0
Total number of TE-AEs within 4 weeks of any infusion	*n*	15	15	9	19	58
Total number of TE-SAEs within 4 weeks of any infusion*	*n*	0	1	1	1	3
Number of patients with:						
At least one TE-AE	*n* (%)	3 (25.0)	7 (53.8)	7 (53.8)	6 (54.5)	23 (46.9)
At least one TE-SAE	*n* (%)	0	1 (7.7)	2 (15.4)	2 (18.2)	5 (10.2)
At least one grade 3 or grade 4 TE-AE	*n* (%)	1 (8.3)	1 (7.7)	2 (15.4)	2 (18.2)	6 (12.2)
At least one related TE-AE	*n* (%)	0	1 (7.7)	1 (7.7)	0	2 (4.1)
At least one related TE-SAE	*n* (%)	0	0	0	0	0
At least one TE-AE leading to study discontinuation	*n* (%)	0	0	0	0	0
At least one TE-AE leading to treatment discontinuation	*n* (%)	0	0	0	0	0
At least one TE-AE leading to treatment interruption	*n* (%)	1 (8.3)	0	1 (7.7)	0	2 (4.1)
At least one TE-SAE with outcome of death	*n* (%)	0	0	0	0	0
At least one TE-AE within 4 weeks of any infusion	*n* (%)	3 (25.0)	7 (53.8)	6 (46.2)	4 (36.4)	20 (40.8)
At least one TE-SAE within 4 weeks of any infusion	*n* (%)	0	1 (7.7)	1 (7.7)	1 (9.1)	3 (6.1)

*Study primary end point.

With regard to the primary end point, three patients experienced a TE-SAE within 4 weeks of administering an infusion, corresponding to one patient in each of the laromestrocel treatment groups. These TE-SAEs were: (1) grade 3 anemia, 5 days after the third infusion (group 3, 25 M × 4); (2) grade 4 lumbar radiculopathy, 19 days after the fourth infusion (group 2, 25 M × 1, after placebo infusion); and (3) grade 4 acute respiratory failure, 28 days after the fourth infusion (group 4, 100 M × 4). All SAEs were related to pre-existing conditions, none of these events was assessed as related to study product, and all resolved. Thus, based on a medical evaluation, the primary end point was met.

Based on the pre-specified statistical safety analysis, the primary end point was met; the lower confidence limit of each laromestrocel group overlapped with the upper confidence limit for group 1 (placebo), indicating a lack of a statistically significant difference in the TE-SAE rate between group 1 (placebo) and any of the laromestrocel groups within 4 weeks after infusion (95% CI for group 1, 0–26.5%; group 2 and group 3, 0.2–36.0%; and group 4, 0.2–41.3%).

With regard to other safety events, the most frequent AEs were COVID-19 and urinary tract infections (Table 2), there were no deaths on the study and no TE-AEs leading to study or treatment discontinuation. No clinically meaningful changes were observed in hematology, chemistry, coagulation or urinalysis parameters. Overall, 23 patients presented with 83 TE-AEs (18 in group 1 (placebo), 21 in group 2 (25 M × 1), 20 in group 3 (25 M×4) and 24 in group 4 (100 M × 4)). Five patients out of 49 presented with six TE-SAEs (one in group 2 (25 M × 1), two in group 3 (25 M × 4) and three in group 4 (100 M × 4)). Two patients had three TE-AEs (one in group 2 (25 M×1) and two in group 3 (25 M × 4)) that were assessed as related to the investigational product (IP) (chills and headache). Four patients experienced six infusion interruptions during the course of the study, none of which was associated with AEs. There were no hypersensitivities or infusion-related reactions observed during the study. Additionally, there were no events of ARIAs (ARIA-H or ARIA-E), including clinically asymptomatic microhemorrhages detected in any study patient. Overall, laromestrocel was safe and well tolerated in the study population, in both single and multiple dosing regimens.

### Secondary outcomes

**Fig. 2:**
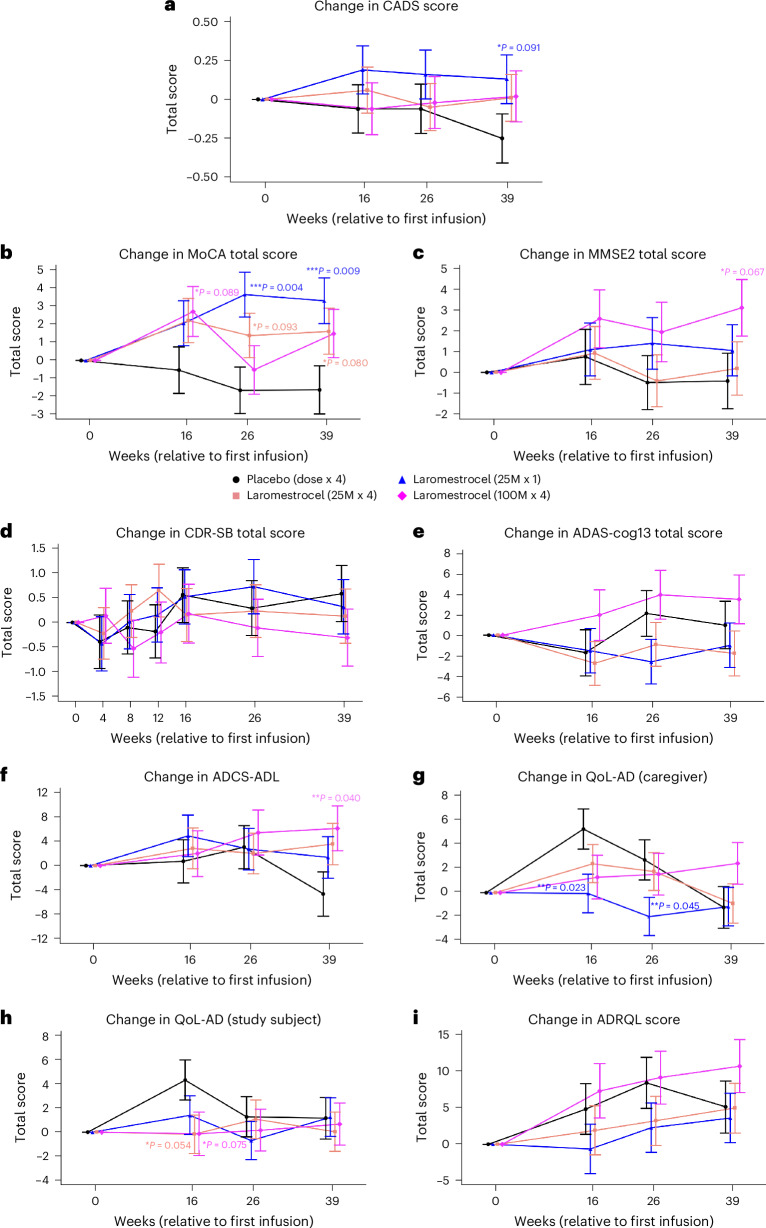
Clinical assessments. Groups 1–4 are color coded: group 1 (placebo; black), group 2 (25 M × 1; blue), group 3 (25 M × 4; salmon) and group 4 (100 M × 4; magenta). Two-sided *P* values are associated with testing for statistically significant differences in least-squares (LS) means between laromestrocel and placebo obtained from a mixed model for repeated measures (MMRM) (F-statistic). No adjustment for multiple comparisons was made. Statistical significance was pre-set at *P* < 0.10 for the CADS scores. For CADS, the placebo group showed a trending decline in CADS scores at 39 weeks (**a**), while treatment groups showed numerical improvement relative to placebo, reaching statistical significance at 39 weeks (group 2, *P* = 0.091; *n* = 11) compared to placebo (*n* = 10). Statistical significance was met for MoCA at 39 weeks versus placebo (*n* = 10) (group 2, *P* = 0.009; *n* = 11) (**b**) as well as for combined groups 2–4, *P* = 0.015; *n* = 32). MMSE-2 (group 4, *P* = 0.067, *n* = 10) (**c**) and CDR-SB (group 4, *P* = 0.270; *n* = 10) (**d**) scores showed numerical improvement for all treatment groups at week 39 compared to placebo (*n* = 10), while ADAS-cog13 measures (combined groups 2–4, *P* = 0.780; *n* = 33) were not statistically different from placebo (*n* = 10) (**e**). **f**–**i**, For QoL measures, statistical significance was met with group 4 for ADCS-ADL at 39 weeks (group 4, *P* = 0.04; *n* = 10) compared to placebo (*n* = 10) (**f**), while group 2 (*P* = 0.228, *n* = 12) and group 3 (*P* = 0.103, *n* = 11) showed numerical improvement relative to placebo (*n* = 10). Numerical improvement was observed in group 4 at 39 weeks for QoL-AD (caregiver) (*P* = 0.142, *n* = 10) compared to placebo (*n* = 10) (**g**), but no improvement was found in QoL-AD (study participant) scores (combined groups 2–4, *P* = 0.797, *n* = 33) compared to placebo (*n* = 10) (**h**) while a numeric trend towards improvement was found in group 4 (*P* = 0.275, *n* = 10) at 39 weeks for ADRQL scores compared to placebo (*n* = 10) (**i**). Error bars represent ±s.e.m.

The pre-specified secondary end point of this study was change from baseline (CFB) to week 39 in a novel Composite Alzheimer’s Disease Score (CADS) in laromestrocel treated groups versus placebo. The CADS was calculated using z-scores for the Alzheimer’s Disease Cooperative Study Activities of Daily Living (ADCS-ADL), Clinical Dementia Rating scale sum of boxes (CDR-SB), Alzheimer’s Disease Assessment Scale-cognitive subscale 13 (ADAS-Cog-13) and left hippocampal volume via MRI, and is similar to other composite score approaches used to measure AD progression, such as the ADCOMS^9,30^, Integrated AD Rating Scale (iADRS)^31^ and Global Statistical Tests (GST)^32^. As depicted in Fig. 2a, the progressive decline of the CADS score (disease worsening) evident in the placebo group was attenuated in both group 2 (25 M × 1) and a combined group consisting of the patients in the active-treatment groups (group 2, 25 M × 1; group 3, 25 M × 4; and group 4, 100 M × 4; hereafter referred to as ‘combined treatment groups 2–4’) compared to placebo, providing provisional support for a slowing of disease progression (group 2 (*n* = 11) versus placebo (*n* = 10) difference 0.38, 95% CI −0.06–0.82; *P* = 0.091; pre-specified *P* < 0.1 as a positive outcome due to a small sample size) (Supplementary Table 3). When evaluated using the per-protocol groups, the CADS was positive for both group 2 (difference 0.39, 95% CI −0.06–0.85, *P* = 0.086; *n* = 11) and group 4 (change 0.44, 95% CI −0.07–0.95, *P* = 0.09; *n* = 7) compared to placebo (*n* = 9).

### Exploratory outcomes

All patients underwent serial cognitive and function clinical assessments using the Montreal Cognitive Assessment (MoCA), mini-mental state evaluation (MMSE-2), CDR-SB and ADAS-Cog-13 (refs. ^33,34^). All laromestrocel groups showed a trend toward improvement in the MoCA (Fig. 2b and Supplementary Table 4) and MMSE-2 (Fig. 2c and Supplementary Table 5) scores compared to placebo (*n* = 10) at week 39, reaching statistical significance for the MoCA in group 2 (25 M × 1; *P* = 0.009; *n* = 11) and for the combined treatment effect group (groups 2–4, *P* = 0.015; *n* = 32). There was a trend toward improvement for MMSE-2 in group 4 (100 M × 4) at 39 weeks (*P* = 0.067; *n* = 10) but treatment did not significantly impact the CDR-SB (Fig. 2d) or ADAS-Cog13 (Fig. 2e).

The ADCS-ADL scores for the high-dose (group 4) and combined treatment groups 2–4 showed improvement at week 39 compared to placebo (*n* = 10), reaching statistical significance for group 4 (100 M × 4, *P* = 0.040; *n* = 10), with trending improvement for the other laromestrocel doses (Fig. 2f and Supplementary Table 6). Quality of life (QoL) as assessed by the caregiver for QoL-AD numerically improved for patients in group 4 relative to group 1 (placebo) (Fig. 2g) but failed to improve as assessed by the study patient (Fig. 2h). No treatment effect compared to placebo was observed at week 39 for laromestrocel treatment groups in the Alzheimer’s disease related quality of life (ADRQL) score (Fig. 2i).

**Fig. 3:**
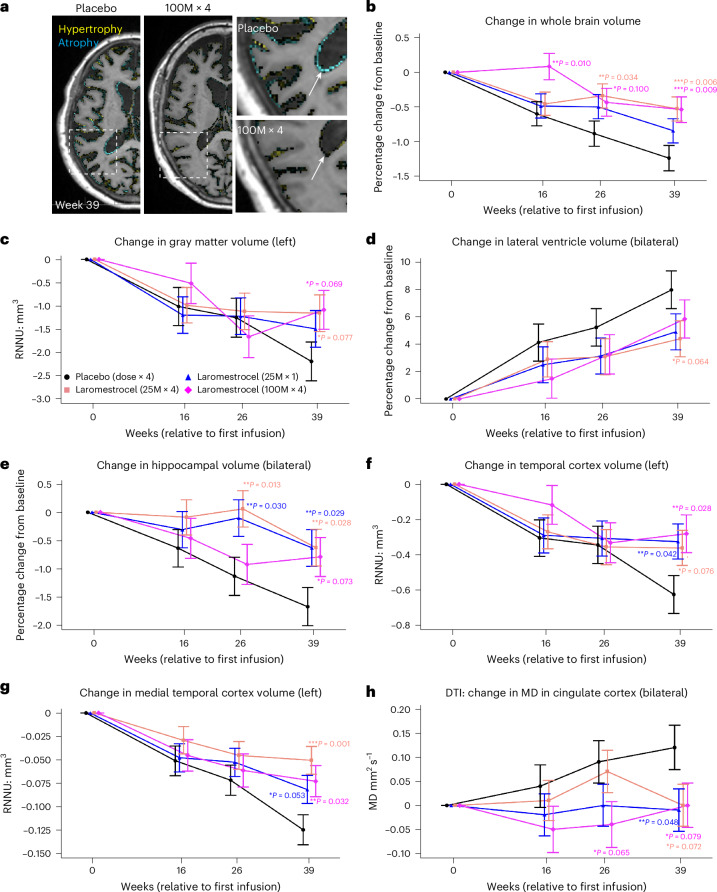
Volumetric MRI assessments of AD patients treated with laromestrocel versus placebo. Two-sided *P* values are associated with testing for statistically significant differences in LS means between laromestrocel and placebo obtained from a MMRM (F-statistic). No adjustment for multiple comparisons was made. **a**, vMRI images depicting changes in brain volume in group 1 (placebo) versus group 4 (high repeated dose) at week 39 (increase, yellow; decrease, blue). Insets in **a** are higher magnification regions of the boxed regions for groups 1 and group 4. **b**, Whole brain volume steadily decreased by ~1.2% in the placebo group during the 39-week trial period, whereas treatment groups showed statistically significant slowing of whole brain atrophy (group 3, *P* = 0.006, *n* = 11; group 4, *P* = 0.009, *n* = 10) compared to placebo (*n* = 10). **c**, Gray matter volume (left) declined in placebo by about 2.2% at week 39, whereas treatment group 4 (*P* = 0.069, *n* = 10) showed numerical improvement compared to placebo (*n* = 10). **d**, Lateral ventricles enlarged in placebo over time, but all treatment groups trended toward improvement at all time points, compared to placebo (*n* = 10), reaching statistical significance for group 3 (right ventricle) at week 39, (*P* = 0.042; *n* = 11). **e**, Bilateral hippocampal volume similarly decreased by approximately 1.6% in placebo (*n* = 10) while treatment groups showed less decline (group 2, *P* = 0.029, *n* = 11; group 3, *P* = 0.028, *n* = 11). **f**, Left temporal cortex showed progressive decline in volume in the placebo group (*n* = 10), but partial rescue with group 2 (*P* = 0.042; *n* = 11) and group 4 (*P* = 0.028; *n* = 10). **g**, Left medial temporal cortex also showed progressive decline in volume in the placebo group (*n* = 10) while treated groups showed slower decline (group 3, *P* = 0.001; *n* = 11; group 4, *P* = 0.032; *n* = 10). Units are indicated as raw non-normalized units ratio to intracranial volume (×10^−^^3^). **h**, Patients with AD receiving placebo showed increasing cingulate cortex diffusion tensor imaging (DTI) values for mean diffusivity (MD) indicating increasing inflammation and disease progression, whereas all groups receiving laromestrocel showed numerically improved MD at 39 weeks, reaching statistical significance for group 2; *P* = 0.048; *n* = 11. Error bars represent ±s.e.m.

Brain atrophy in AD involves multiple brain regions and begins relatively early in disease progression, affecting broad areas of the temporal, occipital, parietal and frontal lobes, and notably the hippocampus, up to 8 years before AD diagnosis^35–38^. In our study, volumetric MRI revealed progressive atrophy at 39 weeks in the placebo group, affecting multiple brain regions and whole brain volume, accompanied by increased ventricular size (Fig. 3). Laromestrocel ameliorated this decline in all treatment groups relative to placebo (Fig. 3). Representative volumetric MRI (vMRI) images depict changes in brain volume in group 1 versus group 4 at week 39 (increase, yellow; decrease, blue) (Fig. 3a). While the 25 M × 1 treatment group (group 2) showed a nonstatistically significant reduction in whole brain atrophy versus the placebo group, both 25 M × 4 and 100 M × 4 groups (groups 3 and 4) showed statistically significant reduction in whole brain atrophy compared to placebo (*n* = 10) by week 39 (by up to 57% for group 3, *P* = 0.006, *n* = 11; group 4, 0.009, *n* = 10) (Fig. 3b). Patients receiving laromestrocel from the combined treatment groups 2–4 (*n* = 32) exhibited 48.4% slower progression of whole brain atrophy at 39 weeks compared to placebo (*n* = 10; *P* = 0.005). Gray matter showed numerical improvement relative to placebo by week 39 (Fig. 3c). Ventricular enlargement, a characteristic feature of progressive AD decline, was observed in placebo (group 1) at week 39. Ventricular enlargement was numerically reduced in the laromestrocel combined treatment groups 2–4 (*n* = 32) by 34% for the left ventricle; (*P* = 0.097) and bilaterally for combined treatment groups 2–4 (*n* = 32) compared to placebo (*n* = 10; 37%, *P* = 0.066) (Fig. 3d) and reached statistical significance for the right ventricle in group 3 (*n* = 11) at week 39 (49%, *P* = 0.042) as well as for the combined treatment groups 2–4 (*n* = 32) compared to placebo (*n* = 10; 40%, *P* = 0.044).

Significantly reduced bilateral hippocampal atrophy was observed for groups 2 and 3 (*P* = 0.029, *n* = 11 and *P* = 0.028, *n* = 11 respectively) at week 39 compared to placebo (*n* = 10), with numerical improvement for group 4 (*P* = 0.073; *n* = 10) (Fig. 3e). Combined laromestrocel treatment groups 2–4 also exhibited reduced left, right, and bilateral hippocampal atrophy by 62%, *P* = 0.021, 53%, *P* = 0.073 and 59%, *P* = 0.013, respectively, versus placebo. The left temporal cortex showed a clear decline in volume at week 39 in group 1 (placebo; *n* = 10), whereas treatment groups 2 and 4 demonstrated statistically significant slowing of decline compared to placebo (*P* = 0.042, *n* = 11 and *P* = 0.028, *n* = 10, respectively) and group 3 trended toward improvement (*P* = 0.076, *n* = 11) (Fig. 3f). The left medial temporal cortex also showed progressive improvement at week 39 versus placebo; while reduced atrophy in group 2 did not reach statistical significance, the higher-dosage groups 3 and 4 demonstrated significant reduction in atrophy relative to placebo (*n* = 10) (group 3, *P* = 0.001, *n* = 11; group 4, *P* = 0.032, *n* = 10) (Fig. 3g). Significantly reduced atrophy was also observed for the right medial temporal cortex for group 3 (*P* = 0.019; *n* = 11) and trending for the combined treatment groups 2–4 compared to placebo (*n* = 10) by week 39 (*P* = 0.064; *n* = 32).

**Extended Data Fig. 1:**
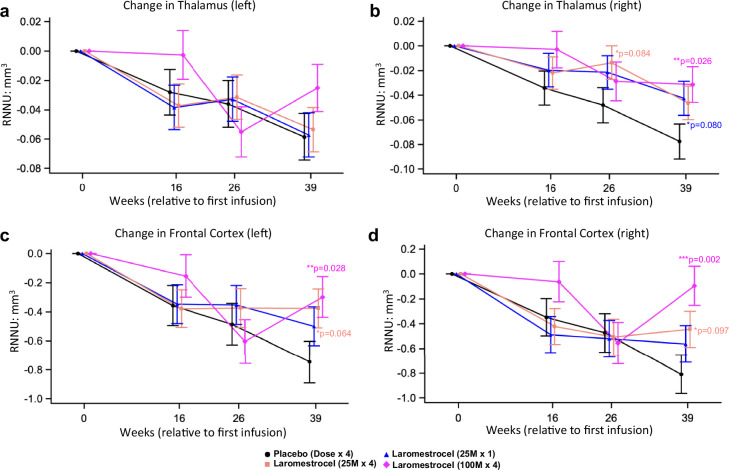
Volumetric MRI assessments of thalamus and frontal cortex of AD patients treated with laromestrocel vs placebo. Two-sided p values are associated with testing for statistically significant differences in LS means between laromestrocel and placebo obtained from a MMRM (F-statistic). No adjustment for multiple comparisons was made. Statistically significant slowing of right thalamus atrophy compared with placebo (N = 10) was observed in Group 4 at week 39 (p = 0.026; N = 10), with numeric slowing in Groups 2 and 3, while no statistically significant change was observed in left thalamic volume (**a**, **b**). Group 4 showed statistically significant slowing of frontal cortical atrophy on both the left (p = 0.028) and right sides (p = 0.002; N = 10) compared with placebo (N = 10) (**c**, **d**). Error bars represent ± SEM.

Notably, the rates of parietal and occipital cortical atrophy were not significantly reduced. Cingulate cortex, striatum and left thalamus similarly showed no significant change compared to placebo, but right thalamus showed statistically significant reduction in atrophy in group 4 (*P* = 0.026, *n* = 10) (Extended Data Fig. 1a,b). Frontal cortex atrophy was also significantly ameliorated by week 39 (left hemisphere: *P* = 0.028; right hemisphere: *P* = 0.002) in group 4 (100 M × 4; *n* = 10), but not in groups 2 and 3 (25 M × 1 and 25 M × 4; Extended Data Fig. 1c,d) compared to placebo (*n* = 10).

Diffusion tensor imaging (DTI) is used to index tissue structure and neuroinflammation and has been a valuable tool in assessing AD brain pathology^39^. In our study, DTI (mean diffusivity; MD) indicated a potential for increased inflammation in AD placebo at 39 weeks compared to baseline (*P* = 0.011; *n* = 10). In contrast, MD was reduced in all laromestrocel treatment groups in the cingulate cortex, compared to placebo (*n* = 10), reaching statistical significance for group 2 (p = 0.048; *n* = 11; Fig. 3h).

**Fig. 4:**
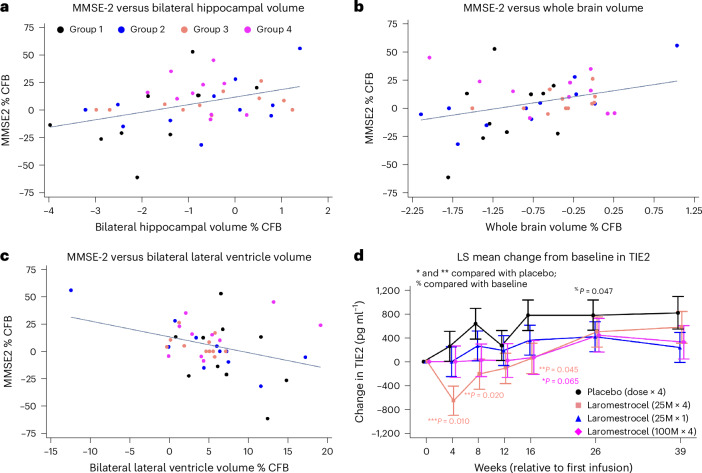
Reduction of bilateral hippocampal, whole brain atrophy correlates with improved MMSE scores. **a**,**b**, Pearson correlation analysis showing correlation between bilateral hippocampal (**a**) and whole brain (**b**) atrophy reduction and improved MMSE-2 scores at week 39 (*R* = 0.41; *P* = 0.0075; *n* = 42 and *R* = 0.35; *P* = 0.0227; *n* = 42, respectively). Correlation of reduced bilateral ventricular enlargement with improved MMSE-2 scores was also evident (*R* = −0.35; *P* = 0.0213; *n* = 42) (**c**). Blood serum collected at the indicated time points was analyzed by Meso Scale Discovery (MSD) for TIE2 (**d**). Two-sided *P* values are associated with testing for statistically significant differences in LS means between laromestrocel and placebo obtained from a MMRM (F-statistic). No adjustment for multiple comparisons was made. Soluble TIE2 levels are represented as raw CFB and were reduced in treatment groups compared to placebo, particularly during the treatment period (to week 16) and reached statistical significance in group 3 at week 4 (*P* = 0.010; *n* = 13), week 8 (*P* = 0.020; *n* = 12) and week 16 (*P* = 0.045; *n* = 12). TIE2 levels were significantly increased in the placebo group (*n* = 11) at week 26 compared to baseline (*P* = 0.047; *n* = 11). Error bars represent ±s.e.m.

We examined blood serum biomarkers to gain insight into the mechanisms of action of laromestrocel in AD. Tyrosine kinase with immunoglobulin and epidermal growth factor homology domains (TIE2), the cognate receptor for angiopoietins 1 and 2, is expressed by endothelial cells, activates pro-angiogenic and anti-inflammatory downstream signaling pathways^40^, and can be degraded into a soluble form (sTIE2) that is released into the bloodstream^41^. We found that sTIE2 levels rose progressively in the placebo group compared to baseline (*P* = 0.047 at week 26; *n* = 11), indicating cell-surface receptor shedding and therefore likely inactivation. Laromestrocel produced a statistically significant reduction in serum levels of sTIE2 relative to placebo for group 3 (25 M × 4) at week 4 (*P* = 0.01; *n* = 12), week 8 (*P* = 0.02; *n* = 12) and week 16 (*P* = 0.045; *n* = 12) (Fig. 4d). sTIE2 levels also trended lower in groups 2 and 4 during the treatment period. sTIE2 levels increased back toward placebo values by weeks 26–39 in all treatment groups.

### Post hoc analysis

We hypothesized that patients with the greatest improvement in vMRI measures would also show the most improvement in cognitive scores. To determine whether reduced brain atrophy correlated with improved clinical scores, we performed a Pearson correlation analysis of week 39 data in all study patients. The change in bilateral hippocampal volume (*R* = 0.41; *P* = 0.008; *n* = 42), whole brain volume (*R* = 0.35; *P* = 0.023; *n* = 42) and bilateral lateral ventricle volume (*R* = −0.35; *P* = 0.0213; *n* = 42), all correlated significantly with change in MMSE-2 scores (Fig. 4a–c).

## Discussion

AD is a disorder of neurocognition with a major unmet medical need. The recent introduction of the disease-modifying class of anti-amyloid monoclonal antibody treatments has ushered in new hope that the natural history of AD could be favorably impacted^13^. Although this class of medicine is now approved, other classes of treatment are still needed. Here we present a phase 2a study of a novel therapeutic class of AD therapy – cell-based therapy that possesses provascular and anti-inflammatory properties^11^. This study provides additional support for the safety profile of this treatment previously seen in a phase 1 study, indicates safety of both single and multiple dosing, and provides preliminary indications of efficacy^27^. The study findings support the potential for laromestrocel to improve both cognitive function and QoL in mild AD. Furthermore, detailed brain-imaging studies reveal potential improvements compared to placebo in brain architecture with laromestrocel administration.

A single previous trial of human umbilical cord derived MSCs explored intracerebroventricular injection in patients with AD. The efficacy of this approach remains to be established and this route of administration was associated with AEs such as fever and headache^42^. The present study further confirms the absence of ARIAs even in individuals who are ApoE carriers, the group previously shown to be at highest risk for ARIAs^14^. Moreover, while both the previous and current studies—limited to 1 year or less—show improvements in some but not all indices of cognitive function and QoL, we hypothesize that longer treatment and longer follow-up times could reveal greater impact.

Statistically significant improvements in brain atrophy were not usually visible at week 16, 4 weeks after the final laromestrocel treatment, but statistically significant changes became evident with subsequent follow-up visits, particularly at week 39. The reduction in brain atrophy was significant on a whole-brain level and was enhanced in several brain regions. Areas that showed the greatest improvements by week 39 included the temporal lobes including the hippocampus—areas that show early atrophy progression and disease pathology in AD^43^—as well as the frontal lobes. It is unclear why these areas showed the greatest benefit; it could indicate a regional bias in the vascular or anti-inflammatory effects of laromestrocel.

Laromestrocel is not expected to directly target β-amyloid or p-Tau, but instead is aimed at the neurovascular and inflammatory components of AD and related disorders. Because we found no evidence for ARIAs, infusion with laromestrocel could indicate a potentially new class of treatment with a favorable safety profile compared to existing anti-amyloid antibody treatments, potentially complimentary to other therapeutic strategies. Moreover, we speculate that laromestrocel expresses protective neurovascular and anti-inflammatory effectors that have the potential to help offset ARIAs in a combination treatment. In addition, we found a correlation of performance on the cognitive scales with hippocampal volume preservation in our study population. Reduced neuroinflammation was suggested via DTI imaging (MD) in the cingulate cortex, an area that shows volume loss in early AD^44,45^, consistent with one of the principal mechanisms of action of laromestrocel. Previous evidence indicates that MSCs and their potential therapeutic effectors (for example exosomes) are active in animals and humans for at least 7–10 days^46–49^, but in the present study the data suggest a potential for a more durable therapeutic benefit. Similar findings of sustained physiologic/clinical improvements have also been observed in studies of MSC administration to patients with congestive heart failure^50,51^ and aging frailty^29^.

We sought evidence of vascular target engagement, and found a statistically significant increase in levels of circulating sTIE2 in the placebo group that were offset by laromestrocel. Consistent with previous studies, the reduced TIE2 levels we observed in blood serum could reflect a decrease in the degradation and ‘shedding’ of the extracellular portion of the TIE2 receptor after cleavage by metalloproteases, which are reported to be upregulated in AD^41,52–54^. Indeed, the increased TIE2 levels we observed at week 26 are supportive of a role for TIE2 degradation in the pathology of AD. In principle, our findings are consistent with a provascular/anti-inflammatory effect of laromestrocel, mediated by circulating secreted factors including but not limited to protein mediators and exosomes^48,55^ in preserving TIE2 signaling in brain vascular endothelial cells^56^. This has the potential to preserve BBB function and improve the brain’s ability to cope with amyloid- and tau-related disease mechanisms. The temporal delay in observing rescue of regional and global atrophy is consistent with an acute but long-lasting effect during the treatment period. The effects of laromestrocel thus might improve neurovascular unit functioning and slow neuroinflammation enough to temper the declining trajectory of neuronal death and atrophy over time during AD progression. It remains unknown whether anti-inflammatory effects in AD could be due to direct anti-inflammatory effectors produced by laromestrocel or indirect due to improved clearance of amyloid^57^ through preserved BBB function. New studies are planned to distinguish between these possibilities.

Not all clinical and imaging biomarker results showed clear dose responses, but repeated dosing (groups 3 and 4) produced better responses than single dosing (group 1) in 27 of 35 total clinical and vMRI measures, and the highest dosage group (group 4) produced the best response in 15 of these, indicating a bias toward increasing effect sizes with higher-dose regimens. On a clinical level, caregiver variability could explain some noise in the data, but on the other hand, vMRI data could be considered more precise. Other contributing factors to data variability, apart from the small sample size, could include variances in the potency of different lots of laromestrocel, as potency may depend partly on the bone-marrow donor. Further research on defining potency of laromestrocel is underway^11,12,25^.

This study had limitations that warrant mentioning. First, there was a relatively small sample size with a high proportion of patients of Hispanic ethnicity and some variables such as patient education level were not ascertained. Second, the 39-week study duration was relatively short for trials of disease-modifying agents in the early AD patient population, and longer studies are warranted. Additionally, while global statistical testing principles support using a composite end point in small studies for AD, the CADS used here is not a validated composite end point and it should be assessed with future studies.

Taken together, the findings of this study suggest that treatment of patients with mild AD with laromestrocel is safe, administered in both single and multiple dosing regimens. New studies using laromestrocel in the treatment of mild AD will examine the safety and efficacy profile of extended treatment durations and follow-up periods with greater statistical power, and we hypothesize longer treatment duration would slow AD progression further. The safety results presented here, coupled with signals of potential efficacy, support ongoing clinical development of this novel class of therapeutics for AD.

## Methods

### Study design and enrollment

The CLEAR-MIND study entitled ‘Lomecel-B Effects on Alzheimer’s disease: A Randomized, Double-Blinded, Placebo-Controlled Phase 2a Trial’ (ClinicalTrials.gov registration: NCT05233774) was conducted using our trial protocol under US FDA IND no. 16524, which is available in the trial master file. Institutional Review Board (IRB) approval was granted by Western-Copernicus Group Institutional Review Board (WCG IRB) on 24 November 2021. The following items were approved at that time: Lomecel-B Drug Brochure (31 August 2023) Protocol v.2.0 (9 November 2021) and Template Consent Form. The primary goal of the study was to demonstrate the safety of intravenous delivery of single- or multiple-dose laromestrocel (Lomecel-B) administered to patients with mild AD. The secondary objective of the study was to identify efficacy signals in single- or multiple-dose laromestrocel versus placebo in the patient population. Efficacy was measured in three domains: cognitive function, QoL and brain architecture measured with vMRI. Patient enrollment occurred at ten clinical centers in South Florida from Martin County to Miami-Dade County between 29 December 2021 and 21 November 2022, with final follow-up on 21 August 2023. All patients provided written consent on the Western IRB-approved protocol. An independent Data and Safety Monitoring Board (DSMB) was responsible for safety and oversight, and recommended study continuation after each planned meeting. Randomization was performed electronically using block sizes of four, equally allocated, with each site receiving separate blocks. Randomization was additionally stratified by sex. Patients were randomized using a 1:1:1:1 ratio into each of four parallel treatment groups. The clinical research organization (CRO), Biorasi, generated the random allocation sequence and assigned patients to intervention groups, while patients were enrolled by the clinical sites. Group 1 patients (*n* = 12) received four infusions of placebo on day 0, weeks 4, 8 and 12. Group 2 patients (*n* = 13) received an infusion of laromestrocel at a dose of 25 × 10^6^ cells (25 million) on day 0, followed by placebo infusions at weeks 4, 8 and 12. Group 3 patients (*n* = 13) received four infusions of laromestrocel 25 M on day 0, weeks 4, 8 and 12. Group 4 patients (*n* = 11) received four infusions of laromestrocel at a dose of 100 × 10^6^ cells (100 million) on day 0, weeks 4, 8 and 12. Stopping rules were used to indicate boundaries requiring DSMB discussion and to assist the DSMB in its duties. The stopping rules were based on continuous monitoring for repeated testing for SAEs using a Pocock-type boundary. We chose to consider the treatment group safe if the proportion of patients with unexpected and possibly related SAEs within 4 weeks of any infusion was 10% or less. Three SAEs occurred during the study and were unrelated to the treatment. The stopping rules were not triggered during the study and the trial was completed.

### Laromestrocel and placebo sourcing and manufacturing

Laromestrocel is an investigational allogeneic adult human MSC therapy, consisting of a suspension of human bone-marrow-derived MSCs^27,29,48,58^. Laromestrocel is obtained from bone-marrow aspirates from the iliac crest of unrelated and human leukocyte antigen (HLA)-unmatched healthy adult donors (ages 18–45 years). Marrow donor characteristics and cell count are provided in Supplementary Table 13. All bone-marrow collections are regulated under 21 CFR 1271, Human Cells, Tissues, and Cellular and Tissue-Based Products (HCT/Ps). All donors are screened in accordance with 21 CFR 1271 Subpart C Donor Eligibility. The material is shipped to Longeveron for processing under aseptic conditions for further processing in a GMP facility. Laromestrocel MSCs were isolated from bone marrow using density gradient separation.

**Extended Data Fig. 2:**
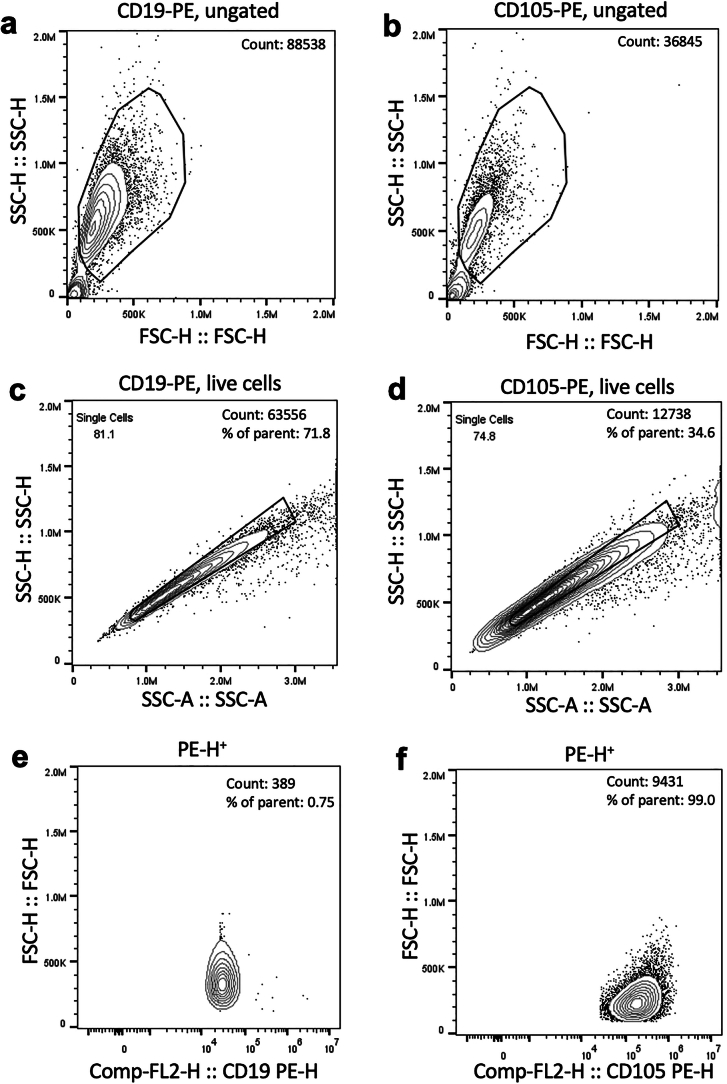
Example flow cytometry workflow for CD19 (negative marker) and CD105 (positive marker) for identity assessment of laromestrocel. Ungated cells (**a, b**) were subcategorized through detection of live cells using the live/dead marker 7-AAD (**c, d**). Single stained cells were distinguished by gating 0.01% of non-stained cells (**e, f**). Laromestrocel lots must be positive for cell surface markers, CD105, CD90, and CD73 ( ≥ 95%). Laromestrocel must be negative ( ≤ 2%) for CD45, CD11b, CD19, and ≤5% for CD34. Abbreviations: SSC-H, side scatter height; FSC-H, forward scatter height; SSC-A, side scatter area.

Laromestrocel is culture expanded in complete medium (α-minimum essential medium (Gibco), containing FBS (Corning)) and grown to approximately 85–90% confluency in multilayer vessels. At each passage, laromestrocel is trypsinized, pooled, subjected to cell count and viability assessments and multilayer vessels are then seeded. All passages utilize the same complete medium. All passages must have cell viability ≥70%. At collection, conditioned medium (supernatant) samples are collected and frozen at −80 °C, then laromestrocel is trypsinized, pooled, subjected to cell count/viability assessment and filled at specific concentrations into CS50 cryobags in a suspension containing a cryoprotectant composed of Hespan, HSA and dimethylsulfoxide (DMSO). Laromestrocel is frozen utilizing-controlled rate freezers and stored at ≤−135 °C in vapor-phase LN_2_ freezers. Each lot of laromestrocel is tested to confirm MSC identity and to ensure all critical quality attributes meet product specifications (Supplementary Table 13). If all specifications are met, the cryopreserved product is released. Identity confirmation is performed via immunophenotyping via flow cytometry. Laromestrocel lots must be positive for cell-surface markers, CD105, CD90 and CD73 (≥95%). Laromestrocel must be negative (≤2%) for CD45, CD11b, CD19, HLA-DR isotype HLA-DR (MHC II cell-surface receptor) and (≤5%) for CD34 (Extended Data Fig. 2). Laromestrocel must be negative for *Mycoplasma*, adventitious viruses via culture on three cell types and negative for Parvo B19, HIV 1, HIV 2, hepatitis B, hepatitis C, HTLV 1, HTLV 2, CMV and EBV by PCR, pass USP 71 sterility with no growth and have post-thaw viability ≥70%.

At the time of infusion, the IP is prepared for administration by Longeveron or a qualified third-party facility. As such, the cryobag containing laromestrocel cryopreserved drug product is thawed in a 37 ± 1 °C water bath. The thawed cell suspension is diluted with medium containing PlasmaLyte-A supplemented with human serum albumin and transferred to an appropriate container. A sample of the cell suspension is counted to determine cell count and viability. The viability must be greater than or equal to 70%. The calculated number of viable cells required to deliver the dose are resuspended and the cell suspension is added to the infusion bag for administration to the patient. The prepared laromestrocel final formulation ready for infusion is maintained at 2–8 °C in a container validated to hold refrigerated temperatures for up to 48 h. Stability testing is performed on all lots of laromestrocel to ensure that infusion product meets viability criteria for a minimum of 8 h. The formulated product for administration is released for patient use following gram stain and endotoxin testing in addition to the cell count and viability.

### Flow cytometry

The identity of MSCs was confirmed by performing immunophenotyping through flow cytometry according to standard methods. In brief, cells were thawed using thaw medium; cell counts and viability were analyzed using a cell counter (NucleoCounter; NC-200; ChemoMetec). Cells were washed with flow staining buffer (cat. no. 00422226, Thermo Fisher) and centrifuged. After centrifugation, cell pellets were resuspended in flow buffer. For the blocking step, cells were added to each tube with appropriate antibodies and incubated in the dark at 2–4 °C. Cells were labeled with live/dead dye; 7-AAD (cat. no. 559925 or cat. no. 00-6993-50; Invitrogen lots 3115592, 1095376 and 2238457); negative controls included: FITC IgG1 (cat. no. 556649, clone MOPC-21, lots 7072907, 7179618, 7165880 and 8274649), PE IgG1 (cat. no. 556650, clone MOPC-21, lots 7165880, 9024919 and 1060595), PCP5.5 IgG1 (cat. no. 552834, clone MOPC-21, lots 9282777 and 1193612 or cat. no. 400149, lot B272763 or cat. no. 400251, lot B236966), APC IgG1 (cat. no. 550854, clone MOPC-21, lots 7215834, 7215839, 9038519 and 2005498). The live/dead and all isotype control IgG1 antibodies were used undiluted at 20 μl. All antibodies were used undiluted and were purchased from BD Biosciences or BioLegend. The cells were labeled with cell-surface marker antibodies for CD105 PE (cat. no. 560839, clone 266, lots 9155633 and 8137932, 5 μl), CD90 FITC (cat. no. 555595 or cat. no. 328108, clone 5E10, lots B2053317, B241990, B205312 and B291322, 5 μl), CD73 APC (cat. no. 560847 or cat. no. 344006, lot B252653 or cat. no. 344005, lot B218129, clone AD2, 5 μl), CD45 FITC (cat. no. 555482 or cat. no. 560976, clone HI30, lots 9077614, 1285678, 7058663 and 8232661, 20 μl), CD34 PE (cat. no. 560941, clone 581, lots 7033536, 203612 and 8221866, 20 μl), CD19 PE (cat. no. 555413 or cat. no. 561741clone HIB19, lots 8081579, 6124709 and 6124705, 20 μl), CD11b PE (cat. no. 561001, clone ICRF44, lots 6251566 and 0066711, 20 μl) and HLA-PC5.5 (cat. no. 560652, clone G46-6, lots 7152686, 8159976, 30984 and 1193612, 5 μl). Unstained cells served as an additional experimental control. After the incubation step, cells were washed with flow cytometry staining buffer solution and centrifuged. Cell pellets were resuspended in flow cytometry staining buffer. All samples were run in triplicate. All data were collected on the CytoFLEX V5-B5-R3 flow cytometer using CytExpert software (Beckman Coulter) and analyzed by FlowJo (Tree Star) (Extended Data Fig. 2).

### Exploratory testing of laromestrocel conditioned medium for bioactive effectors

Conditioned medium samples acquired at the time of collection were tested for the presence of potential bioactive secreted proteins, including TIMP2 (by ELISA; Abcam cat. no. 100653, using lots 1012154 and 1065326 following the manufacturer’s instructions) and VEGF-A, VEGF-D, PlGF, TIE2, interleukin (IL)-2, IL-4, IL-6, IL-8, IL-10, IL-12/p70, IL-13, IL-1β, TNF and IFNγ (V-PLEX and V-PLEX Plus Angiogenesis Panel 1 (Human) kits; cat. no. 15190G, 15190D and V-PLEX Plus Proinflammatory Panel 1 Human kit cat. no. K15049G-1 from MSD), according to the manufacturer’s instructions. TIMP2 concentrations in the samples (in ng ml^−1^) were further normalized to the cell density at the time of laromestrocel collection (cells per ml) and represented as ng per cell. MSD analyses were performed using an MSD instrument (MESO QuickPlex SQ 120MM) and DISCOVERY WORKBENCH v.4.0 software. (Supplementary Table 14).

### Patient population

Eligible patients had a clinical diagnosis of mild AD in accordance with the National Institute of Aging and the Alzheimer’s Association criteria at the time of enrollment. Additionally, a positron emission tomography (PET) scan using a US FDA-approved tracer (for example, florbetapir-fluorine-18 (AMYViD), Vizamyl or Neuraceq) was required, consistent with the diagnosis of AD. Further criteria included APOe4 positivity, MMSE-2 score of 18–24, age 60–85 years, presence of an adult caregiver willing and able to participate in the study and accompany the patient to all study visits and Brain MRI consistent with AD, excluding any other brain abnormalities that can cause dementia (such as stroke, mass lesions or hydrocephalus). Patients were excluded with any other neurodegenerative disease, history of seizure disorder or evidence of previous macro hemorrhages.

### Study procedures and timelines

Screening activity was performed within 6 weeks before the first infusion treatment and included informed consent, medical history and physical examination collection, ECG, clinical assessments including MMSE-2 (ref. ^59^), ADAS-Cog-13 (ref. ^60^), CDR-SB^61^, QoL-AD^62^, ADCS-ADL^63^, ADRQL^63^, Neuropsychiatric Inventory (NPI)^29^, specimen collection for safety labs, biomarker samples and urine sample analysis. Informed consent was obtained for all study participants. Participants were not compensated for their participation in the study. Only self-reported sex categories were collected during the study and included the categories ‘male’ and ‘female’. This was used as a stratification factor as AD affects women disproportionately compared to men. No other sex analysis was performed at screening. Imaging via MRI and amyloid PET scan completed the screening procedure^64^.

The baseline visit activity was performed within 4 weeks before the first infusion and included a review of the medical history for any changes, MoCA testing, re-administration of the cognitive testing except for MMSE-2 as well as specimen collection. Screening and baseline were separated by at least 2 weeks. All visit dates were planned with respect to the first infusion, defined as time zero (0). Infusions were conducted on day 0, weeks 4, 8 and 12. During the infusion visits, laromestrocel or placebo (PlasmaLyte containing HSA) were administered via peripheral intravenous infusion over 40 min. Before infusion a review of concomitant medications and AEs was conducted as well as administration of the CDR-SB and specimen collection. Follow-up visits were completed at weeks 16, 26 and 39, which included clinical assessment and review, ECG, patient and caregiver assessments, specimen collection and MRI brain scans.

### Study end points

#### Primary end point

To ensure safety, the patients were monitored after each infusion of laromestrocel (for example, on day 0 and weeks 4, 8 and 12). The safety assessments were measured by evaluating the incidence of any AEs and SAEs within 4 weeks of infusion; any changes in blood and urine sample analysis; and any ARIAs or clinically asymptomatic microhemorrhages as revealed by MRI in the study. The primary end point was the difference from placebo in the rate of TE-SAEs occurring within 4 weeks of any infusion. The TE-SAE criteria employed in this trial captured all SAEs between the first infusion and end of study.

#### Secondary end point

To analyze the efficacy of treatment, a CADS assessment was calculated at baseline and weeks 16, 26, and 39. The CADS was evaluated at each visit by averaging equally weighted *z*-scores for the following components: ADCS-ADL total score, CDR-SB total score (inverted), overall ADAS-Cog-13 total score (inverted) and left hippocampal volume (normalized for intracranial volume). The estimated marginal mean CADS at week 39 was used as our secondary end point in comparing each laromestrocel group versus placebo as well as the combined laromestrocel groups versus placebo.

#### Exploratory end points

The neurocognitive, neuropsychiatric, QoL, activities of daily living and Canadian Study of Health and Aging Clinical Frailty Score (CSHA-CFS) assessments described above were performed at screening, baseline and weeks 16, 26 and 39 to assess the CFB. In addition, biomarkers were assessed as exploratory efficacy measurement: serum-based biomarkers related to potential provascular and anti-inflammatory activities of laromestrocel, as well as AD progression, were measured at screening, baseline, day 0 and weeks 4, 8, 12, 16, 26 and 39. A vascular-related biomarker, sTIE2, was evaluated based on previous studies that implicate laromestrocel as having the potential to inhibit the cleavage of TIE2 from the cell membrane, and as such, offsetting its inactivation^41,52–54^. sTIE2 was analyzed using an MSD electrochemiluminescence immunoassay machine (1300 MESO QuickPlex SQ120) and angiogenesis panel kit (MSD; cat. no. K15190D-1). Quantification was completed using a MSD V-Plex Assay with duplicate samples per patient visit.

Brain volumetry was performed via MRI at screening and weeks 16, 26 and 39, to assess volumetric changes in the hippocampus, overall brain size, ventricular volume and other brain structures, each normalized for intracranial volume (refs). DTI via MRI was conducted at screening and weeks 16, 26 and 39, to assess for changes in neuroinflammation. DTI scalars include fractional anisotropy (FA)^65^, mean diffusivity (MD), axial diffusivity (AD) and radial diffusivity (RD)^66^. Furthermore, the neurocognitive, neuropsychiatric, QoL, activities of daily living and Canadian Study of Health and Aging Clinical Frailty Score assessments were performed at screening (excluding MoCA), baseline (excluding MMSE-2) and weeks 16, 26 and 39 to assess the CFB.

### vMRI and PET imaging

Independent central imaging services were supported by Bioclinica, a Clario company. Bioclinica developed imaging protocols, trained the imaging centers, performed quality assurance on all images and completed the blinded data analysis. Brain MRIs were performed using 3T (Tesla) scanners, two imaging centers were used by the ten clinical centers, one MRI scanner at each location was used for the entire trial. Whole and regional brain volumes were calculated via MRI, including hippocampal and ventricular volume, each normalized for intracranial volume, and collected at screening and weeks 16, 26 and 39. The DTI indices (FA, AD, RD and MD) were calculated from the native DTI acquisition. Whole-brain vMRI scans were used in this study, with atlas-based subregion identification. For vMRI and atrophy analysis, all 3DT1 scans were N4 bias-corrected, registered to a 256-mm^3^ template space and isotropically resampled to achieve a voxel size of 1 mm^3^. Preprocessed 3DT1 scans were then analyzed in FreeSurfer 6 to generate baseline regional volume measures of all available cortical/subcortical, white matter and ventricular regions. For atrophy measures, follow-up imaging time points were registered with baseline scans to a log-symmetric midway space for tensor-based morphometry analysis. All volume and atrophy measures are reported as either % CFB or as regional/intracranial volume ratio using raw non-normalized units (mm^3^) as applicable. For 3DT1 MPRAGE, parameters included sagittal acquisition with 176 slices, a field of view of 240 × 240 mm; slice thickness of 1.2 mm; TR/TE of 1,800ms/2.49 ms; TI = 900 ms; flip angle of 10 degrees; and a matrix size of 192 × 192.

PET^64^ imaging was collected at screening using a US FDA-approved tracer (for example, florbetapir-fluorine-18 (AMYViD), Vizamyl or Neuraceq) consistent with the diagnosis of AD. A previous positive PET scan was allowed with sponsor approval.

### DTI imaging

DTI parameters were axial spin echo; echo planar imaging acquisition with 80 slices; field of view = 232 × 232 mm; slice thickness 2 mm; phase encode direction, AP, TR/TE, min/min (but still T2-weighted, dependent on scanner performance); matrix size, 116 × 116; bandwidth, 1,486 Hz/Px; directions, 30; b-value = 1,000 s mm^−2^; and 2 b0 averages. In addition, a DTI b0 reverse sequence with identical parameters (including TR/TE/BW) but with phase encode direction, PA.

A standardized DTI sequence (2 mm^3^ voxel size, 30 directions, b-value = 1,000 s mm^−2^) was deployed to each site in conjunction with a reverse-phase encode DTI sequence (blip down) with identical contrast parameters (TR/TE/bandwidth) for distortion correction. Scans were received and quality controlled to ensure adherence to approved acquisition settings. Pre-processing included eddy and motion correction that was performed using a linear affine transformation on all gradient images with the baseline image of b = 0 s mm^−2^. The reverse encoding DTI image (blip down acquisition) was employed for correcting the distortions in the main DTI image (blip up acquisition) using DRBUDDI, which is based on symmetric diffeomorphic registration to estimate the distortion field. Diffusion tensors were then computed using a standard LS approximation and the scalar maps of FA, MD, axial diffusivity and RD were derived from the tensor image. The FA maps were then registered to the FreeSurfer processed 3DT1 image in standard 256 × 256 × 256 space and the deformation was applied to remaining maps (MD, AD and RD). Finally, the FA image was registered to The Johns Hopkins University (JHU) atlas’s FA image using advanced normalization tools (ANTs). Symmetric normalization (SyN) deformable registration and deformation were applied to all the maps. This allowed for a seamless overlay of cortical, subcortical and white matter labels for regional value extraction. For each subject, the registrations and overlays were checked manually and reprocessed or rejected in case of failures^65,66^.

### Statistical analysis

All statistical analyses were conducted using SAS software (v.9.4). Safety analyses were performed on the ITT population, based on the actual treatment received. For the primary end point, Clopper–Pearson exact confidence intervals were calculated for percentage of patients with any SAEs within 4 weeks after an infusion for all treatment groups (group 1 (placebo), group 2 (25 M × 1), group 3 (25 M × 4) and group 4 (100 M × 4)) as well as the laromestrocel pooled 2, 3 and 4 group (25 M × 1, 25 M × 4, 100 M × 4) and the laromestrocel pooled 3 and 4 group (25 M × 4, 100 M × 4). Additional safety parameters (laboratory, vital signs and ECGs) were summarized descriptively by treatment group and assessment time.

The secondary end point, CADS, was calculated by combining *z*-scores for CFB (to study end point) values for multiple assessments: ADCS-ADL, CDR-SB, ADAS-Cog-13 and left hippocampal volume (normalized for intracranial volume). Each component comprised 25% of the final score (each equally weighted). Inverted measures of the ADAS-Cog-13 and CDR-SB were used to match directionality of the other measures with respect to improvement/decline changes. Change from baseline in CADS was statistically analyzed with an MMRM analysis. The model included fixed effects for visit, treatment group (4 level variable), visit by treatment interaction, sex and baseline value of the outcome parameter. ‘Patient’ was included as a random effect. LS means, s.e. and 95% CIs for mean CFB were obtained from the model for each treatment group at each visit, including the pooled laromestrocel treatment group. The LS-mean differences, s.e., 95% CIs and two-sided *P* values for the differences between treatment groups were obtained for each active-treatment group (groups 2, 3 and 4) relative to placebo (group 1) at each visit. The pooled laromestrocel treatment group effect relative to placebo was obtained from the same model. Because this study was a hypothesis generating study, for CADS, if the two-sided *P* value for the difference was <0.1, then the difference was considered statistically significant and further studies would be warranted. No adjustments for multiple comparisons were performed and no primary or secondary outcome data were excluded.

Powering was performed based on the results of a completed phase 1 study of laromestrocel^27^. The study was not powered to detect a statistically significant difference in CADS. This study was originally designed to detect a difference in MMSE-2 of 3.87 points at 39 weeks between each laromestrocel treatment group and placebo at 85% power. During the course of the study, the key secondary end point was amended in the trial protocol to the CADS score. A protocol amendment was approved by the US FDA before unblinding, guided by the practice of adopting composite end points for AD; however, assuming a two-sided α of 0.1, the power to detect a 50% slowing of disease progression based on CADS comparing three active arms combined (*n* = 36) versus placebo (*n* = 12) was 36% at 6 months. Effect sizes as small as 0.2–0.3 in CADS may be considered clinically meaningful. No interim analyses were conducted.

The ITT population included all randomized patients who received at least one full or partial dose of IP (any infusion of either laromestrocel or placebo). The mITT population included all patients who were randomized and received at least one full or partial dose of the IP (any infusion of either laromestrocel or placebo) and completed at least one post-baseline efficacy assessment (biomarker data or cognitive test).

Similar MMRM analyses were conducted for the exploratory efficacy end points of ADAS-cog-13, MMSE-2, ADCS-ADL, CDR-SB, MoCA, Neuropsychiatric Inventory (NPI), QoL-AD (both caregiver and study participant), ADCS-ADL, ADRQL, brain volumetry (via MRI) and DTI.

### Reporting summary

Further information on research design is available in the Nature Portfolio Reporting Summary linked to this article.

## Online content

Any methods, additional references, Nature Portfolio reporting summaries, source data, extended data, supplementary information, acknowledgements, peer review information; details of author contributions and competing interests; and statements of data and code availability are available at 10.1038/s41591-025-03559-0.

## Supplementary information

Supplementary Information Supplementary Tables 1–14, with captions.

Reporting Summary

## Data Availability

All requests for data access should be sent to the corresponding authors, B.G.R. or J.M.H. The minimum dataset, without individual patient data, used for the primary, secondary and exploratory conclusions, may be shared under a data-use agreement for IRB-approved research. Requests will be considered and responded to within 1 month of receipt. The trial protocol and statistical analysis plan under US FDA IND no. 16524 can be shared upon academic or research request.

## Code availability

SAS v.9.4 was used for all analyses included in the manuscript. SAS code for the minimum dataset is on file and can be made available under a code use agreement for IRB-approved research. Requests for this code should be sent to the corresponding authors, B.G.R. or J.M.H. Code for volumetric MRI and DTI image analysis was generated by Clario and is not publicly available; special requests for this code can be directed to Clario.
