# Supplementary material for: Allogeneic mesenchymal stem cell therapy with laromestrocel in mild Alzheimer’s disease: a randomized controlled phase 2a trial
Source: Nat Med. 2025 Mar 10;31(4):1257–66. doi: 10.1038/s41591-025-03559-0 (PMC12003194; doi:10.1038/s41591-025-03559-0)
Supplement: Supplementary file 1 — Supplementary Tables 1–14, with captions. [file 41591_2025_3559_MOESM1_ESM.pdf]

nature medicine

Supplementary information https://doi.org/10.1038/s41591-025-03559-0

Allogeneic mesenchymal stem cell therapy with laromestrocel in mild Alzheimer's disease: a randomized controlled phase 2a trial

In the format provided by the authors and unedited

Table S1: Volumetric MRI Baseline Characteristics (mITT).

Parameter: mean (SD)	Group 1 (Placebo) (N=12)	Group 2 (25Mx1) (N=12)	Group 3 (25Mx4) (N=13)	Group 4 (100Mx4) (N=11)	P-value
Whole Brain	683.112 (33.988)	709.373 (58.649)	710.017 (36.605)	706.158 (58.819)	0.468
Grey Matter - Left	192.165 (8.095)	198.084 (15.542)	198.444 (12.602)	198.578 (13.702)	0.553
Grey Matter - Right	192.757 (9.160)	198.103 (16.609)	199.345 (13.035)	199.142 (15.055)	0.612
Lat. Ventricle - Left	14.696 (4.560)	12.441 (5.820)	17.013 (9.584)	13.444 (7.026)	0.399
Lat. Ventricle - Right	13.357 (3.718)	11.117 (4.711)	14.297 (8.345)	12.750 (7.307)	0.630
Hippocampal - Bilateral	5.077 (0.528)	5.117 (0.879)	5.284 (0.689)	5.423 (0.542)	0.595
Hippocampal - Left	2.472 (0.280)	2.529 (0.402)	2.632 (0.294)	2.649 (0.223)	0.456
Hippocampal - Right	2.605 (0.279)	2.588 (0.497)	2.652 (0.435)	2.774 (0.340)	0.682
Temporal Cortex - Left	34.232 (2.557)	36.308 (5.052)	36.515 (4.179)	36.829 (3.671)	0.387
Medial Temporal Cortex - Left	5.805 (0.623)	6.071 (0.910)	6.124 (0.677)	6.545 (0.669)	0.129
Medial Temporal Cortex - Right	6.095 (0.651)	5.969 (1.051)	6.212 (0.775)	6.455 (0.767)	0.540
Cingulate Cortex - Left	6.000 (0.487)	6.456 (0.635)	6.263 (0.822)	6.319 (0.728)	0.415
Cingulate Cortex - Right	5.757 (0.593)	5.944 (0.535)	6.050 (0.908)	5.966 (0.900)	0.801
Thalamus - Left	4.644 (0.346)	4.644 (0.395)	4.674 (0.458)	4.654 (0.509)	0.998
Thalamus - Right	4.644 (0.270)	4.524 (0.535)	4.597 (0.372)	4.653 (0.509)	0.876
Frontal Cortex - Left	51.726 (2.616)	53.550 (3.529)	53.939 (3.589)	52.778 (4.196)	0.422
Frontal Cortex - Right	51.424 (2.608)	53.285 (4.055)	53.520 (3.800)	52.396 (4.445)	0.507

Abbreviations: SD=Standard Deviation. All parameters were normalized to intra-cranial volume prior to analysis. Group 1: Placebo (Dose x 4). Group 2: Lomecel-B (25M x 1). Group 3: Lomecel-B (25M x 4). Group 4: Lomecel-B (100M x 4). Values represent means (SD) for normalized regional volume/intracranial volume ratio x 10^3.

Table S2: Inclusion and exclusion criteria

**Inclusion Criteria**	At screening, each patient ***must*** meet all the following criteria to be enrolled.<br>1. Provide written informed consent.<br>2. Be 60 – 85 years of age at signing of the Informed Consent Form.<br>3. Clinical diagnosis of mild Alzheimer's disease in accordance with the NIA-AA criteria at the time of enrollment.<br>4. MMSE-2 score of 18 – 24.<br>5. Body weight of 40 – 150 kg.<br>6. Has an adult caregiver who meets all of the following criteria.<br>a. Provides written informed consent to participate on the trial (reporting on patient observations).<br>b. Either lives with the patient or sees the patient for at least 2 hours/day for at least 3 days/week.<br>c. Is willing and able to participate in the study and agrees to accompany the patient to each study visit.<br>d. Is able to read, understand, and speak the designated language at the study site.<br>7. Brain MRI consistent with AD, excluding any other brain abnormalities which can cause dementia (such as strokes, mass lesions, hydrocephalus).<br>8. A PET scan using an FDA-approved tracer (e.g., AMYViD, Vizamyl, or Neuraceq) consistent with the diagnosis of AD. A prior positive PET scan will be allowed with Sponsor approval.<br>9. Living in the community, includes assisted living facilities (but excluding long-term care nursing facilities).
**Exclusion Criteria**	At screening, each patient ***must not*** meet any of the following criteria to be enrolled.<br>1. Diagnosed with frontotemporal dementia (FTD), dementia due to Acquired Immunodeficiency Syndrome (AIDS), Creutzfeldt-Jakob disease (CJD), Lewy Bodies dementia (LBD), Progressive Supranuclear Palsy (PSP), multiple cerebral infarctions, or normal pressure hydrocephalus.<br>2. Any other neurodegenerative disease.<br>3. History of a seizure disorder.<br>4. Evidence of a prior macrohemorrhage; at least 4 cerebral microhemorrhages (regardless of anatomical location or diagnostic characterization as "possible" or "definite"); or at least 1 area of superficial siderosis.<br>5. Unwillingness or inability to have MRI scans (no contrasting agent will be used), or condition that contraindicates MRI, such

	as the presence metallic objects in the eyes, skin, or heart.<br>6. Any condition that contraindicates PET with a beta-amyloid tracer.<br>7. Significant intestinal malabsorption surgery, e.g., gastric bypass.<br>8. If serum B12 level is below normal range, follow-up is required to rule out B12 deficiency-related cognitive decline.<br>9. Clinically abnormal free T4 or thyroid-stimulating hormone (TSH).<br>10. Resting blood oxygen saturation <93%.<br>11. Resting systolic blood pressure >180 mm Hg, or diastolic blood pressure >110 mm Hg.<br>12. Regularly (> 4 weeks) using high-doses of corticosteroids or other steroidal anti-inflammatory medication (e.g., Prednisone) on a regular basis, with the exception of steroidal nasal sprays, asthma inhalers, topical steroids, and hormonal-replacement therapy.<br>13. Regularly (> 4 weeks) using anti-cytokine antibody or targeting therapy, e.g., anti-TNF-α.<br>14. Be an organ transplant recipient or have active or expected future listing for any organ/tissue transplant while scheduled to be on trial, except for corneal, bone, skin, ligament, or tendon.<br>15. Diagnosed with malignancy within the past 2 years, with the exception of curatively treated basal cell carcinoma, squamous cell carcinoma, melanoma in situ, or cervical carcinoma.<br>16. Known hypersensitivity to dimethyl sulfoxide (DMSO).<br>17. Test positive for hepatitis B virus surface antigen, viremic hepatitis C virus, HIV, or syphilis.<br>18. Any condition that is projected to limited life expectancy to < 14 months.<br>19. Be pregnant, nursing, or of childbearing potential while not practicing effective contraception.<br>20. Be currently participating or have participated in any other investigational therapeutic or device trial within the previous 30 days to screening. Exclusion for participation in any other trial over 30 days to screening is up to the PI's discretion.<br>21. In the opinion of the investigator, the patient has any other illness or condition that: may compromise the patient's safety, compliance, or ability to successfully complete the study; may compromise the validity of the study; or otherwise, should exclude the patient from enrollment.

Table S3: Composite Alzheimer's Disease Score (CADS) results. Least squares (LS) means, as well as LS mean difference from placebo, values are reported for Weeks 16, 26, and 39 in a mixed model repeated measures analysis.

	Group 1 (Placebo)	Group 2 (25Mx1)	Group 3 (25Mx4)	Group 4 (100Mx4)
**Week 16**<br>LS Mean (SE)<br>95% CI<br>n	 -0.06 (0.155)<br>(-0.37, 0.25)<br>11	 0.19 (0.155)<br>(-0.12, 0.50)<br>11	 0.06 (0.149)<br>(-0.24, 0.35)<br>12	 -0.06, (0.167)<br>(-0.39, 0.28)<br>9
**Week 16**<br>LS Mean difference (SE)<br>95% CI<br>P-value<br>n		 0.25 (0.218)<br>(-0.18, 0.69)<br>0.251<br>11	 0.12 (0.215)<br>(-0.31, 0.55)<br>0.584<br>12	 0.01, (0.229)<br>(-0.45, 0.46)<br>0.981<br>9
**Week 26**<br>LS Mean (SE)<br>95% CI<br>n	 -0.06 (0.158)<br>(-0.38, 0.25)<br>10	 0.16, (0.157)<br>(-0.15, 0.47)<br>11	 -0.05 (0.151)<br>(-0.35, 0.25)<br>11	 -0.02 (0.167)<br>(-0.35, 0.31)<br>9
**Week 26**<br>LS Mean difference (SE)<br>95% CI<br>P-value<br>n		 0.22, (0.220)<br>(-0.22, 0.66)<br>0.314<br>11	 0.01 (0.219)<br>(-0.42, 0.45)<br>0.948<br>11	 0.05 (0.231)<br>(-0.41, 0.50)<br>0.846<br>9
**Week 39**<br>LS Mean (SE)<br>95% CI<br>n	 -0.25 (0.158)<br>(-0.56, 0.07)<br>10	 0.13 (0.157)<br>(-0.18, 0.44)<br>11	 0.01 (0.151)<br>(-0.29, 0.32)<br>11	 0.02 (0.164)<br>(-0.30, 0.35)<br>10
**Week 39**<br>LS Mean difference (SE)<br>95% CI<br>P-value<br>n		 0.38 (0.221)<br>(-0.06, 0.82)<br>0.091<br>11	 0.26 (0.219)<br>(-0.18, 0.70)<br>0.238<br>11	 0.27 (0.228)<br>(-0.19, 0.72)<br>0.243<br>10

Table S4: Montreal cognitive assessment (MoCA) results. Least squares (LS) mean, as well as LS mean difference from placebo, values are reported for Weeks 16, 26, and 39 in a mixed model repeated measures analysis.

	Group 1 (Placebo)	Group 2 (25Mx1)	Group 3 (25Mx4)	Group 4 (100Mx4)
**Week 16**<br>LS Mean (SE)<br>95% CI<br>n	 -0.54 (1.280)<br>(-3.09, 2.00)<br>11	 2.05 (1.238)<br>(-0.41, 4.52)<br>12	 2.20 (1.228)<br>(-0.24, 4.65)<br>12	 2.70, (1.38)<br>(-0.05, 5.45)<br>9
**Week 16**<br>LS Mean difference (SE)<br>95% CI<br>P-value<br>n		 2.60 (1.785)<br>(-0.96, 6.15)<br>0.150<br>12	 2.75 (1.765)<br>(-0.77, 6.26)<br>0.123<br>12	 3.25, (1.885)<br>(-0.51, 7.00)<br>0.089<br>9
**Week 26**<br>LS Mean (SE)<br>95% CI<br>n	 -1.64 (1.280)<br>(-4.18, 0.91)<br>11	 3.64, (1.238)<br>(1.17, 6.10)<br>12	 1.37 (1.228)<br>(-1.07, 3.82)<br>12	 -0.52 (1.333)<br>(-3.17, 2.14)<br>10
**Week 26**<br>LS Mean difference (SE)<br>95% CI<br>P-value<br>n		 5.27, (1.785)<br>(1.72, 8.82)<br>0.004<br>12	 3.01 (1.765)<br>(-0.51, 6.52)<br>0.093<br>12	 1.12 (1.852)<br>(-2.57, 4.81)<br>0.547<br>10
**Week 39**<br>LS Mean (SE)<br>95% CI<br>n	 -1.62 (1.322)<br>(-4.25, 1.02)<br>10	 3.29 (1.267)<br>(0.77, 5.81)<br>11	 1.61 (1.266)<br>(-0.91, 4.13)<br>11	 1.48 (1.333)<br>(-1.17, 4.14)<br>10
**Week 39**<br>LS Mean difference (SE)<br>95% CI<br>P-value<br>n		 4.90 (1.832)<br>(1.26, 8.55)<br>0.009<br>11	 3.23 (1.821)<br>(-0.40, 6.85)<br>0.080<br>11	 3.10 (1.880)<br>(-0.64, 6.84)<br>0.103<br>10

Table S5: Mini mental state exam (MMSE)-2 results. Least squares (LS) mean, as well as LS mean difference from placebo, values are reported for Weeks 16, 26, and 39 in a mixed model repeated measures analysis.

	Group 1 (Placebo)	Group 2 (25Mx1)	Group 3 (25Mx4)	Group 4 (100Mx4)
**Week 16**<br>LS Mean (SE)<br>95% CI<br>n	 0.75 (1.319)<br>(-1.87, 3.38)<br>10	 1.10 (1.256)<br>(-1.40, 3.61)<br>11	 0.93 (1.265)<br>(-1.59, 3.45)<br>11	 2.56, (1.384)<br>(-0.20, 5.32)<br>9
**Week 16**<br>LS Mean difference (SE)<br>95% CI<br>P-value<br>n		 0.35 (1.812)<br>(-3.26, 3.96)<br>0.848<br>11	 0.17 (1.836)<br>(-3.48, 3.83)<br>0.924<br>11	 1.81, (1.909)<br>(-2.00, 5.61)<br>0.347<br>9
**Week 26**<br>LS Mean (SE)<br>95% CI<br>n	 -0.48 (1.291)<br>(-3.05, 2.09)<br>11	 1.39, (1.229)<br>(-1.06, 3.84)<br>12	 -0.40 (1.240)<br>(-2.87, 2.07)<br>12	 1.93 (1.427)<br>(-0.91, 4.78)<br>8
**Week 26**<br>LS Mean difference (SE)<br>95% CI<br>P-value<br>n		 1.87, (1.774)<br>(-1.66, 5.41)<br>0.294<br>12	 0.08 (1.799)<br>(-3.51, 3.66)<br>0.965<br>12	 2.41 (1.921)<br>(-1.41, 6.24)<br>0.213<br>8
**Week 39**<br>LS Mean (SE)<br>95% CI<br>n	 -0.41 (1.324)<br>(-3.05, 2.23)<br>10	 1.06 (1.229)<br>(-1.39, 3.51)<br>12	 0.20 (1.269)<br>(-2.33, 2.73)<br>11	 3.09 (1.343)<br>(-0.42, 5.77)<br>10
**Week 39**<br>LS Mean difference (SE)<br>95% CI<br>P-value<br>n		 1.47 (1.796)<br>(-2.11, 5.05)<br>0.416<br>12	 0.61 (1.843)<br>(-3.06, 4.28)<br>0.742<br>11	 3.50 (1.883)<br>(-0.25, 7.26)<br>0.067<br>10

Table S6: ADCS-ADL results. Least squares (LS) means, as well as LS mean difference from placebo, values are reported for Weeks 16, 26, and 39 in a mixed model repeated measures analysis.

	Group 1 (Placebo)	Group 2 (25Mx1)	Group 3 (25Mx4)	Group 4 (100Mx4)
**Week 16**<br>LS Mean (SE)<br>95% CI<br>n	 0.69 (3.528)<br>(-6.34, 7.71)<br>11	 4.87 (3.410)<br>(-1.92, 11.66)<br>12	 2.83 (3.356)<br>(-3.85, 9.51)<br>12	 1.97, (3.766)<br>(-5.53, 9.46)<br>9
**Week 16**<br>LS Mean difference (SE)<br>95% CI<br>P-value<br>n		 4.18 (4.895)<br>(-5.56, 13.93)<br>0.395<br>12	 2.14 (4.866)<br>(-7.54, 11.83)<br>0.661<br>12	 1.28, (5.152)<br>(-8.97, 11.54)<br>0.804<br>9
**Week 26**<br>LS Mean (SE)<br>95% CI<br>n	 3.05 (3.528)<br>(-3.97, 10.07)<br>11	 2.70, (3.410)<br>(-4.09, 9.49)<br>12	 2.00 (3.356)<br>(-4.68, 8.68)<br>12	 5.40 (3.683)<br>(-1.94, 12.73)<br>10
**Week 26**<br>LS Mean difference (SE)<br>95% CI<br>P-value<br>n		 -0.35, (4.895)<br>(-10.09, 9.40)<br>0.944<br>12	 -1.05 (4.866)<br>(-10.74, 8.63)<br>0.829<br>12	 2.35 (5.095)<br>(-7.80, 12.49)<br>0.647<br>10
**Week 39**<br>LS Mean (SE)<br>95% CI<br>n	 -4.64 (3.602)<br>(-11.81, 2.53)<br>10	 1.37 (3.410)<br>(-5.42, 8.16)<br>12	 3.53 (3.421)<br>(-3.28, 10.34)<br>11	 6.10 (3.683)<br>(-1.24, 13.43)<br>10
**Week 39**<br>LS Mean difference (SE)<br>95% CI<br>P-value<br>n		 6.01 (4.947)<br>(-3.84, 15.86)<br>0.228<br>12	 8.17 (4.960)<br>(-1.70, 18.04)<br>0.103<br>11	 10.74 (5.145)<br>(0.50, 20.98)<br>0.040<br>10

Table S7: MRI results for whole brain volume. Least squares (LS) means of % change from baseline, as well as LS mean difference from placebo, values are reported for Weeks 16, 26, and 39 in a mixed model repeated measures analysis.

	Group 1 (Placebo)	Group 2 (25Mx1)	Group 3 (25Mx4)	Group 4 (100Mx4)
**Week 16**<br>LS Mean (SE)<br>95% CI<br>n	 -0.60 (0.175)<br>(-0.95, -0.25)<br>11	 -0.49 (0.173)<br>(-0.83, -0.14)<br>11	 -0.46 (0.168)<br>(-0.79, -0.12)<br>12	 0.08 (0.190)<br>(-0.30, 0.46)<br>9
**Week 16**<br>LS Mean difference (SE)<br>95% CI<br>P-value		 0.11 (0.245)<br>(-0.38, 0.60)<br>0.649	 0.14 (0.242)<br>(-0.34, 0.63)<br>0.557	 0.68 (0.259)<br>(0.16, 1.20)<br>0.010
**Week 26**<br>LS Mean (SE)<br>95% CI<br>n	 -0.88 (0.181)<br>(-1.24, -0.52)<br>10	 -0.50 (0.174)<br>(-0.84, -0.15)<br>11	 -0.34 (0.173)<br>(-0.69, 0.00)<br>11	 -0.43 (0.199)<br>(-0.83, -0.04)<br>8
**Week 26**<br>LS Mean difference (SE)<br>95% CI<br>P-value		 0.38 (0.250)<br>(-0.11, 0.88)<br>0.129	 0.54 (0.250)<br>(0.04, 1.04)<br>0.034	 0.45 (0.270)<br>(-0.09, 0.99)<br>0.100
**Week 39**<br>LS Mean (SE)<br>95% CI<br>n	 -1.23 (0.182)<br>(-1.60, -0.87)<br>10	 -0.84 (0.174)<br>(-1.19, -0.50)<br>11	 -0.53 (0.173)<br>(-0.87, -0.18)<br>11	 -0.54 (0.183)<br>(-0.90, -0.17)<br>10
**Week 39**<br>LS Mean difference (SE)<br>95% CI<br>P-value		 0.39 (0.250)<br>(-0.11, 0.89)<br>0.120	 0.71 (0.251)<br>(0.21, 1.21)<br>0.006	 0.70 (0.258)<br>(0.18, 1.21)<br>0.009

Table S8: MRI results for gray matter (left) volume. Least squares (LS) means of raw non-normalized units (mm^3), as well as LS mean difference from placebo, values are reported for Weeks 16, 26, and 39 in a mixed model repeated measures analysis.

	Group 1 (Placebo)	Group 2 (25Mx1)	Group 3 (25Mx4)	Group 4 (100Mx4)
**Week 16**<br>LS Mean (SE)<br>95% CI<br>n	 -1.01 (0.408)<br>(-1.83, -0.20)<br>11	 -1.20 (0.392)<br>(-1.98, -0.42)<br>11	 -0.98 (0.381)<br>(-1.74, -0.22)<br>12	 -0.51 (0.431)<br>(-1.37, 0.35)<br>9
**Week 16**<br>LS Mean difference (SE)<br>95% CI<br>P-value		 -0.18 (0.566)<br>(-1.31, 0.94)<br>0.746	 0.03 (0.560)<br>(-1.09, 1.15)<br>0.959	 0.50 (0.598)<br>(-0.69, 1.69)<br>0.405
**Week 26**<br>LS Mean (SE)<br>95% CI<br>n	 -1.25 (0.419)<br>(-2.09, -0.42)<br>10	 -1.22 (0.393)<br>(-2.00, -0.43)<br>11	 -1.11 (0.390)<br>(-1.89, -0.34)<br>11	 -1.66 (0.447)<br>(-2.55, -0.77)<br>8
**Week 26**<br>LS Mean difference (SE)<br>95% CI<br>P-value		 0.04 (0.572)<br>(-1.10, 1.18)<br>0.951	 0.14 (0.574)<br>(-1.01, 1.28)<br>0.811	 -0.41 (0.617)<br>(-1.64, 0.83)<br>0.513
**Week 39**<br>LS Mean (SE)<br>95% CI<br>n	 -2.19 (0.419)<br>(-3.02, -1.35)<br>10	 -1.49 (0.393)<br>(-2.28, -0.71)<br>11	 -1.16 (0.391)<br>(-1.93, -0.38)<br>11	 -1.08 (0.420)<br>(-1.92, -0.25)<br>10
**Week 39**<br>LS Mean difference (SE)<br>95% CI<br>P-value		 0.70 (0.572)<br>(-0.44, 1.84)<br>0.227	 1.03 (0.576)<br>(-0.11, 2.18)<br>0.077	 1.11 (0.599)<br>(-0.09, 2.30)<br>0.069

Table S9: MRI results for bilateral lateral ventricle volume. Least squares (LS) means of % change from baseline, as well as LS mean difference from placebo, values are reported for Weeks 16, 26, and 39 in a mixed model repeated measures analysis.

	Group 1 (Placebo)	Group 2 (25Mx1)	Group 3 (25Mx4)	Group 4 (100Mx4)
**Week 16**				
LS Mean (SE)	4.11 (1.340)	2.49 (1.307)	2.90 (1.293)	1.49 (1.429)
95% CI	(1.44, 6.78)	(-0.12, 5.09)	(0.32, 5.47)	(-1.36, 4.34)
n	11	11	12	9
**Week 16**				
LS Mean difference (SE)		-1.63 (1.864)	-1.22 (1.858)	-2.62 (1.960)
95% CI		(-5.34, 2.09)	(-4.92, 2.49)	(-6.53, 1.28)
P-value		0.386	0.515	0.185
**Week 26**				
LS Mean (SE)	5.22 (1.366)	3.13 (1.311)	3.08 (1.316)	3.26 (1.430)
95% CI	(2.50, 7.94)	(0.52, 5.74)	(0.46, 5.70)	(0.41, 6.11)
n	10	11	11	8
**Week 26**				
LS Mean difference (SE)		-2.09 (1.885)	-2.14 (1.891)	-1.96 (1.980)
95% CI		(-5.85, 1.66)	(-5.91, 1.63)	(-5.91, 1.99)
P-value		0.271	0.261	0.325
**Week 39**				
LS Mean (SE)	7.95 (1.368)	4.89 (1.311)	4.39 (1.313)	5.83 (1.399)
95% CI	(5.23, 10.68)	(2.27, 7.50)	(1.78, 7.01)	(3.04, 8.62)
n	10	11	11	10
**Week 39**				
LS Mean difference (SE)		-3.07 (1.886)	-3.56 (1.891)	-2.12 (1.960)
95% CI		(-6.83, 0.69)	(-7.33, 0.21)	(-6.03, 1.78)
P-value		0.108	0.064	0.283

Table S10: MRI results for bilateral hippocampal volume. Least squares (LS) means of % change from baseline, as well as LS mean difference from placebo, values are reported for Weeks 16, 26, and 39 in a mixed model repeated measures analysis.

	Group 1 (Placebo)	Group 2 (25Mx1)	Group 3 (25Mx4)	Group 4 (100Mx4)
**Week 16**				
LS Mean (SE)	0.64 (0.328)	-0.31 (0.322)	-0.09 (0.312)	-0.46 (0.354)
95% CI	(-1.29, 0.02)	(-0.95, 0.33)	(-0.71, 0.53)	(-1.17, 0.25)
n	11	11	12	9
**Week 16**				
LS Mean difference (SE)		0.33 (0.458)	0.55 (0.453)	0.18 (0.482)
95% CI		(-0.58, 1.24)	(-0.35, 1.45)	(-0.78, 1.14)
P-value		0.475	0.230	0.714
**Week 26**				
LS Mean (SE)	-1.13 (0.337)	-0.10 (0.324)	0.06 (0.321)	-0.93 (0.354)
95% CI	(-1.80, -0.46)	(-0.75, 0.54)	(-0.58, 0.70)	(-1.63, -0.22)
n	10	11	11	8
**Week 26**				
LS Mean difference (SE)		1.03 (0.466)	1.19 (0.465)	0.21 (0.489)
95% CI		(0.10, 1.96)	(0.26, 2.12)	(-0.77, 1.18)
P-value		0.030	0.013	0.674
**Week 39**				
LS Mean (SE)	-1.67 (0.338)	-0.63 (0.324)	-0.63 (0.321)	-0.79 (0.343)
95% CI	(-2.34, -0.99)	(-1.27, 0.02)	(-1.27, 0.01)	(-1.48, -0.11)
n	10	11	11	10
**Week 39**				
LS Mean difference (SE)		1.04 (0.466)	1.04 (0.466)	0.88 (0.481)
95% CI		(0.11, 1.97)	(0.11, 1.97)	(-0.08, 1.84)
P-value		0.029	0.028	0.073

Table S11: MRI results for left temporal cortex volume. Least squares (LS) means of raw non-normalized units (mm^3), as well as LS mean difference from placebo, values are reported for Weeks 16, 26, and 39 in a mixed model repeated measures analysis.

	**Group 1 (Placebo)**	**Group 2 (25Mx1)**	**Group 3 (25Mx4)**	**Group 4 (100Mx4)**
**Week 16**				
LS Mean (SE)	-0.30 (0.104)	-0.29 (0.100)	-0.27 (0.097)	-0.12 (0.110)
95% CI	(-0.51, -0.10)	(-0.49, -0.09)	(-0.46, -0.08)	(-0.34, 0.10)
n	11	11	12	9
**Week 16**				
LS Mean difference (SE)		0.01 (0.144)	0.03 (0.143)	0.19 (0.153)
95% CI		(-0.27, 0.30)	(-0.25, 0.32)	(-0.12, 0.49)
P-value		0.920	0.810	0.226
**Week 26**				
LS Mean (SE)	-0.34 (0.106)	-0.31 (0.100)	-0.36 (0.099)	-0.33 (0.114)
95% CI	(-0.56, -0.13)	(-0.51, -0.11)	(-0.55, -0.16)	(-0.56, -0.10)
n	10	11	11	8
**Week 26**				
LS Mean difference (SE)		0.04 (0.145)	-0.01 (0.146)	0.01 (0.158)
95% CI		(-0.25, 0.33)	(-0.30, 0.28)	(-0.30, 0.33)
P-value		0.798	0.933	0.937
**Week 39**				
LS Mean (SE)	-0.62 (0.107)	-0.32 (0.100)	-0.36 (0.099)	-0.28 (0.107)
95% CI	(-0.84, -0.41)	(-0.52, -0.13)	(-0.56, -0.16)	(-0.49, -0.07)
n	10	11	11	10
**Week 39**				
LS Mean difference (SE)		0.30 (0.145)	0.26 (0.146)	0.34 (0.154)
95% CI		(0.01, 0.59)	(-0.03, 0.56)	(0.04, 0.65)
P-value		0.042	0.076	0.028

Table S12: MRI results for left medial temporal cortex volume. Least squares (LS) means of raw non-normalized units (mm^3), as well as LS mean difference from placebo, values are reported for Weeks 16, 26, and 39 in a mixed model repeated measures analysis.

	Group 1 (Placebo)	Group 2 (25Mx1)	Group 3 (25Mx4)	Group 4 (100Mx4)
**Week 16**<br>LS Mean (SE)<br>95% CI<br>n	 -0.05 (0.016)<br>(-0.08, -0.02)<br>11	 -0.05 (0.015)<br>(-0.08, -0.02)<br>11	 -0.03 (0.015)<br>(-0.06, 0.00)<br>12	 -0.05 (0.017)<br>(-0.08, -0.01)<br>9
**Week 16**<br>LS Mean difference (SE)<br>95% CI<br>P-value		 0.00 (0.022)<br>(-0.04, 0.05)<br>0.876	 0.02 (0.021)<br>(-0.02, 0.06)<br>0.305	 0.01 (0.024)<br>(-0.04, 0.05)<br>0.808
**Week 26**<br>LS Mean (SE)<br>95% CI<br>n	 -0.07 (0.016)<br>(-0.10, -0.04)<br>10	 -0.05 (0.015)<br>(-0.08, -0.02)<br>11	 -0.05 (0.015)<br>(-0.08, -0.02)<br>11	 -0.06 (0.018)<br>(-0.10, -0.03)<br>8
**Week 26**<br>LS Mean difference (SE)<br>95% CI<br>P-value		 0.02 (0.022)<br>(-0.02, 0.06)<br>0.387	 0.03 (0.022)<br>(-0.02, 0.07)<br>0.234	 0.01 (0.024)<br>(-0.04, 0.06)<br>0.671
**Week 39**<br>LS Mean (SE)<br>95% CI<br>n	 -0.12 (0.016)<br>(-0.16, -0.09)<br>10	 -0.08 (0.015)<br>(-0.11, -0.05)<br>11	 -0.05 (0.015)<br>(-0.08, -0.02)<br>11	 -0.07 (0.017)<br>(-0.11, -0.04)<br>10
**Week 39**<br>LS Mean difference (SE)<br>95% CI<br>P-value		 0.04 (0.022)<br>(0.00, 0.09)<br>0.053	 0.07 (0.022)<br>(0.03, 0.12)<br>0.001	 0.05 (0.024)<br>(0.00, 0.10)<br>0.032

Table S13. Characterization of Lomecel-B production lots: bone marrow donor characteristics, harvest cell count, and MSC identity and release testing results. Abbreviations: LMSC, Lomecel-B; BMI, body mass index; N/A, not available.

**Donor characteristics**		LMSC024	LMSC037	LMSC040	LMSC042	LMSC045	LMSC053	LMSC055	LMSC064
Age:		27	32	31	41	35	21	18	28
Sex:		F	M	F	M	M	M	M	F
BMI (kg/m^2):		22.2	25.1	30.1	32.3	29.3	23	19.9	N/A
Harvest Cell Count:		$2.84x10^9$	$1.24x10^{10}$	$5.31x10^9$	$4.8x10^9$	$5.52x10^9$	$1.15x10^9$	$8.26x10^9$	$5.86x10^9$
**Attribute/Test**	**Acceptance Criteria**								
Viability	≥ 70%	94.2	89.3	87.0	83.0	74.2	80.7	83.3	97.2
CD105	≥ 95%	96.6	99.5	95.8	99.9	99.8	99.93	99.86	98.2
CD90	≥ 95%	99.4	99.6	96.1	100	100	99.93	99.76	98.3
CD73	≥ 95%	99.7	99.5	95.2	100	95.2	100	99.97	99.4
CD34	≤ 5%	0.12	2.78	0.26	2.53	3.68	0.46	0.41	0.18
CD45	≤ 2%	0.12	0.13	0.23	0.23	0.09	0.66	0.65	0.361
CD11b/14	≤ 2%	0.13	0.19	0.54	0.38	0.36	0.92	1.19	1.58
CD19	≤ 2%	0.09	0.08	0.025	0.14	0.13	0.27	0.46	0.334
Endotoxin	≤ 5 EU/mL	≤0.100	≤0.100	≤0.100	≤0.100	≤0.100	≤0.100	≤0.100	≤0.100
Mycoplasma	Negative	Negative	Negative	Negative	Negative	Negative	Negative	Negative	Negative
Sterility	Negative	Negative	Negative	Negative	Negative	Negative	Negative	Negative	Negative
Adventitious Virus Assay	Negative	NA*	NA*	NA*	NA*	Negative	Negative	Negative	Negative
Parvo b19	Negative	NA*	NA*	NA*	NA*	Negative	Negative	Negative	Negative
HIV 1&2	Negative	NA*	NA*	NA*	NA*	Negative	Negative	Negative	Negative
Hepatitis B	Negative	NA*	NA*	NA*	NA*	Negative	Negative	Negative	Negative
Hepatitis C	Negative	NA*	NA*	NA*	NA*	Negative	Negative	Negative	Negative
HTLV 1&2	Negative	NA*	NA*	NA*	NA*	Negative	Negative	Negative	Negative
CMV	Negative	NA*	NA*	NA*	NA*	Negative	Negative	Negative	Negative
EBV	Negative	NA*	NA*	NA*	NA*	Negative	Negative	Negative	Negative

Table S14. Characterization of Lomecel-B production lots: potential bioactive proteins. TIMP2 protein concentration was measured by ELISA and normalized to cell density and represented as ng/cell. Other analytes were measured using MSD. Analytes that showed values below the lower limit of quantification (LLOQ) are marked as ND (not detected). Analytes that were detected above the LLOQ values were further normalized to the cell density and the data are represented as pg/cell. N/A: test sample not available.

Assays for Lomecel-B's Functional Characterization	***Lot numbers***							
Markers	LMSC024	LMSC037	LMSC040	LMSC042	LMSC045	LMSC053	LMSC055	LMSC064
Detection of Human TIMP2 through ELISA								
TIMP2 (ng/cell)	$2.43x10^{-4}$	$1.69x10^{-4}$	$1.71x10^{-4}$	$1.47x10^{-4}$	$2.68x10^{-4}$	$2.81x10^{-4}$	$3.65x10^{-4}$	$1.95x10^{-4}$
Detection of angiogenesis markers through validated multiplex MSD								
VEGF-A (pg/cell)	$2.88x10^{-2}$	$1.34x10^{-2}$	$3.94x10^{-2}$	$2.82x10^{-2}$	$3.27x10^{-2}$	$3.72x10^{-2}$	$5.32x10^{-2}$	$2.76x10^{-2}$
PIGF (pg/cell)	$3.71x10^{-4}$	$2.01x10^{-4}$	$4.10x10^{-4}$	$2.26x10^{-4}$	$1.78x10^{-4}$	$6.17x10^{-4}$	$4.39x10^{-4}$	$7.14x10^{-4}$
VEGF-D (pg/cell)	ND	ND	ND	ND	ND	ND	ND	ND
TIE2 (pg/cell)	ND	ND	ND	ND	ND	ND	ND	ND
Detection of proinflammatory markers through validated multiplex MSD								
IL-6 (pg/cell)	$1.81x10^{-2}$	$0.70x10^{-2}$	N/A	$0.99x10^{-2}$	$1.72x10^{-2}$	$2.41x10^{-2}$	$1.08x10^{-2}$	$0.83x10^{-2}$
IL-8 (pg/cell)	$0.12x10^{-2}$	$0.13x10^{-2}$	N/A	$0.09x10^{-2}$	$0.07x10^{-2}$	$0.40x10^{-2}$	$0.04x10^{-2}$	$0.05x10^{-2}$
IL-2 (pg/cell)	ND	ND	N/A	ND	ND	ND	ND	ND
IL-4 (pg/cell)	ND	ND	N/A	ND	ND	ND	ND	ND
IL-10 (pg/cell)	ND	ND	N/A	ND	ND	ND	ND	ND
IL-12p70 (pg/cell)	ND	ND	N/A	ND	ND	ND	ND	ND
IL-13 (pg/cell)	ND	ND	N/A	ND	ND	ND	ND	ND
IL-1β (pg/cell)	ND	ND	N/A	ND	ND	ND	ND	ND
TNF-α (pg/cell)	ND	ND	N/A	ND	ND	ND	ND	ND
IFN-γ (pg/cell)	ND	ND	N/A	ND	ND	ND	ND	ND
