# Supplementary material for: Allogeneic mesenchymal stem cell therapy with laromestrocel in mild Alzheimer’s disease: a randomized controlled phase 2a trial
Source: Nat Med. 2025 Mar 10;31(4):1257–66. doi: 10.1038/s41591-025-03559-0 (PMC12003194; doi:10.1038/s41591-025-03559-0)
Supplement: Supplementary file 2 — Reporting Summary [file 41591_2025_3559_MOESM2_ESM.pdf]

nature portfolio

Corresponding author(s): Brian G. Rash and Joshua M. Hare

Last updated by author(s): Jan 22, 2025

Reporting Summary

Nature Portfolio wishes to improve the reproducibility of the work that we publish. This form provides structure for consistency and transparency in reporting. For further information on Nature Portfolio policies, see our Editorial Policies and the Editorial Policy Checklist.

Statistics

For all statistical analyses, confirm that the following items are present in the figure legend, table legend, main text, or Methods section.

n/a	Confirmed	
☐	☒	The exact sample size (*n*) for each experimental group/condition, given as a discrete number and unit of measurement
☐	☒	A statement on whether measurements were taken from distinct samples or whether the same sample was measured repeatedly
☐	☒	The statistical test(s) used AND whether they are one- or two-sided<br>*Only common tests should be described solely by name; describe more complex techniques in the Methods section.*
☐	☒	A description of all covariates tested
☐	☒	A description of any assumptions or corrections, such as tests of normality and adjustment for multiple comparisons
☐	☒	A full description of the statistical parameters including central tendency (e.g. means) or other basic estimates (e.g. regression coefficient) AND variation (e.g. standard deviation) or associated estimates of uncertainty (e.g. confidence intervals)
☐	☒	For null hypothesis testing, the test statistic (e.g. *F*, *t*, *r*) with confidence intervals, effect sizes, degrees of freedom and *P* value noted<br>*Give P values as exact values whenever suitable.*
☒	☐	For Bayesian analysis, information on the choice of priors and Markov chain Monte Carlo settings
☐	☒	For hierarchical and complex designs, identification of the appropriate level for tests and full reporting of outcomes
☐	☒	Estimates of effect sizes (e.g. Cohen's *d*, Pearson's *r*), indicating how they were calculated

Our web collection on statistics for biologists contains articles on many of the points above.

Software and code

Policy information about availability of computer code

Data collection	FreeSurfer 6
Data analysis	SAS 9.4 was used for all analyses included in the manuscript. SAS code is on file and can be made available according to our code availability statement (below):<br><br>Code availability<br>SAS version 9.4 was used for all analyses included in the manuscript. SAS code for the minimum dataset is on file and can be made available under a code use agreement for IRB approved research. Requests for this code should be sent to the corresponding authors, BGR or JMH. Code for volumetric MRI and DTI image analysis was generated by Clario and is not publicly available; special requests for this code can be directed to Clario.

For manuscripts utilizing custom algorithms or software that are central to the research but not yet described in published literature, software must be made available to editors and reviewers. We strongly encourage code deposition in a community repository (e.g. GitHub). See the Nature Portfolio guidelines for submitting code & software for further information.

Data

Policy information about availability of data

All manuscripts must include a data availability statement. This statement should provide the following information, where applicable:

- Accession codes, unique identifiers, or web links for publicly available datasets
- A description of any restrictions on data availability
- For clinical datasets or third party data, please ensure that the statement adheres to our policy

Data availability
Please send all requests for data access to the corresponding authors, BGR or JMH. The minimum dataset, without individual patient data, used for the primary, secondary, and exploratory conclusions, may be shared under a data use agreement for IRB approved research. Requests will be considered and responded to within 1 month of receipt. The trial protocol and statistical analysis plan under US FDA IND # 16524 can be shared upon academic or research request.

Research involving human participants, their data, or biological material

Policy information about studies with human participants or human data. See also policy information about sex, gender (identity/presentation), and sexual orientation and race, ethnicity and racism.

Reporting on sex and gender	Sex and gender information was self-reported by study participants and recorded within the EDC. Informed consent was obtained for all study participants. Participants were not compensated for their participation in the study.<br><br>Only the self-reported sex categories were collected during the study and included the categories “male” and “female.” This was used as a stratification factor as AD affects women disproportionately compared to men. No other sex analysis was performed.
Reporting on race, ethnicity, or other socially relevant groupings	Race and ethnicity information was self-reported by study participants and recorded within the EDC.
Population characteristics	A total of 120 patients with a clinical diagnosis of mild AD were screened and 50 were enrolled. Eligible patients had a clinical diagnosis of mild Alzheimer’s Disease in accordance with the National Institute of Aging and the Alzheimer’s Association (NIA-AA) criteria at the time of enrollment. Additionally, a Positron Emission Tomography (PET) scan using a Food and Drug Administration (FDA) -approved tracer (e.g., Florbetapir-fluorine-18 (AMYViD), Vizamyl, or Neuraceq) was required, consistent with the diagnosis of AD. Further criteria included APOe4 positivity, Mini-Mental State Evaluation (MMSE-2) score of 18-24, age 60-85 years, presence of an adult caregiver willing and able to participate in the study and accompany the patient to all study visits and Brain Magnetic Resonance Imaging (MRI) consistent with AD, excluding any other brain abnormalities which can cause dementia (such as stroke, mass lesions or hydrocephalus). Patients were excluded with any other neurodegenerative disease, history of seizure disorder or evidence of prior macro hemorrhages. Sex was used as a covariate in all mixed effects repeated measures models.
Recruitment	Patient enrollment occurred at 10 clinical centers in South Florida from Martin County to Miami-Dade County between December 29, 2021, and November 21, 2022, with final follow-up on August 21, 2023. All patients provided written consent on the Western Institutional Review board- approved protocol. An independent Data and Safety Monitoring Board (DSMB) was responsible for safety and oversight, and recommended study continuation after each planned meeting. There are no known self-selection or other recruitment biases that could impact study results; the study employed a double blind, placebo-controlled, randomized design. Change from baseline in CADS was statistically analyzed with a mixed model for repeated measures (MMRM) analysis. The model included fixed effects for visit, treatment group (4 level variable), visit by treatment interaction, sex, and baseline value of the outcome parameter.
Ethics oversight	The CLEAR-MIND study entitled “Lomecel-B Effects on Alzheimer’s disease: A Randomized, Double-Blinded, Placebo-Controlled Phase 2a Trial” (ClinicalTrials.gov registration: NCT05233774) was conducted using our trial protocol under US FDA IND # 16524, which is available in the trial master file. Protocol granted by U.S. Food and Drug Administration, under IND 16524. IRB approval was granted by WCG IRB on 11/24/2021. The following items were approved at that time: Lomecel-B Drug Brochure (08-31-2023) Protocol Version 2.0 (11-09 2021) and Template Consent Form.

Note that full information on the approval of the study protocol must also be provided in the manuscript.

Field-specific reporting

Please select the one below that is the best fit for your research. If you are not sure, read the appropriate sections before making your selection.

☒ Life sciences ☐ Behavioural & social sciences ☐ Ecological, evolutionary & environmental sciences

For a reference copy of the document with all sections, see nature.com/documents/nr-reporting-summary-flat.pdf

Life sciences study design

All studies must disclose on these points even when the disclosure is negative.

Sample size	Assuming a two-sided alpha of 0.1, the power for the CADS comparing 3 active arms combined (N=36) vs placebo (N=12) at 50% slowing is 0.14 at 3 months or 0.36 at 6 months (insufficient 9-month data). This study was not powered for statistical significance of the CADS endpoint, however effect sizes as small as 0.2-0.3 may be considered clinically meaningful.
Data exclusions	The mITT population was used for all analyses described within the manuscript. The Modified Intention-to-Treat Population (mITT) includes all patients who are randomized and received at least one full or partial dose of the investigational product (any infusion, either Lomecel-B or placebo) and completed at least 1 (one) post-baseline efficacy assessment (biomarker data or cognitive test).patient
Replication	To ensure reproducibility of the findings, the trial firstly implemented a randomized, double-blind, placebo-controlled design. Secondly, for the core prespecified analysis (primary, secondary, key secondary, exploratory analyses, laboratory, and imaging data), Longeveron used a validated data set of tables, figures, and listings (TFL), which was reproduced by two independent biostatisticians at our contracted CRO, Biorasi. We are not aware of any failures in reproducing the results.<br>Additionally, we have implemented several rounds of QC of the manuscript to ensure accuracy of all statistics, figures, and tables, in correspondence with the tables, figures, and listings associated with and contained within the clinical study report. These numbers have been independently verified by multiple individuals at Longeveron, including our clinical biostatisticians. A small number of typeos were found and corrected.
Randomization	This was a placebo-controlled, double-blinded trial. Patients were randomized using a 1:1:1:1 ratio of each active treatment group (Group 2, 3 or 4) and placebo (Group 1). Randomization was performed in block sizes of 4 for each gender, and separate blocks per site.
Blinding	The patient and clinical teams were blinded regarding the treatment arms. Only the Investigational Product (IP) depot was unblinded. As applicable and when possible, biological samples were analyzed and evaluated by blinded staff or an independent third party.

Reporting for specific materials, systems and methods

We require information from authors about some types of materials, experimental systems and methods used in many studies. Here, indicate whether each material, system or method listed is relevant to your study. If you are not sure if a list item applies to your research, read the appropriate section before selecting a response.

Materials & experimental systems

n/a	Involved in the study
☐	☒ Antibodies
☐	☒ Eukaryotic cell lines
☒	☐ Palaeontology and archaeology
☒	☐ Animals and other organisms
☐	☒ Clinical data
☒	☐ Dual use research of concern
☒	☐ Plants

Methods

n/a	Involved in the study
☒	☐ ChIP-seq
☐	☒ Flow cytometry
☐	☒ MRI-based neuroimaging

Antibodies

Antibodies used	Cells were labeled with live/dead dye; 7-AAD (cat# 559925 or cat#00-6993-50; Invitrogen lots#3115592, 1095376, 2238457); negative controls included: FITC IgG1 (cat#556649, clone MOPC-21, lots#7072907, 7179618, 7165880, 8274649), PE IgG1 (cat#556650, clone MOPC-21, lots#7165880, 9024919, 1060595), PCP5.5 IgG1 (cat#552834, clone MOPC-21, lots# 9282777, 1193612 or cat#400149, lot#B272763 or cat#400251, lot#B236966), APC IgG1 (cat#550854, clone MOPC-21, lots#7215834, 7215839, 9038519, 2005498). The live/dead and all isotype control IgG1 antibodies were used undiluted at 20uL. All antibodies were used undiluted and were purchased from BD Biosciences or BioLegend. The cells were labeled with cell surface marker antibodies for CD105 PE (cat#560839, clone 266, lots#9155633, 8137932, 5uL), CD90 FITC (cat#555595 or cat#328108, clone 5E10, lots#B2053317, B241990, B205312, B291322, 5uL), CD73 APC (cat# 560847, or cat#344006, lot# B252653 or cat#344005, lot# B218129, clone AD2, 5uL), CD45 FITC (cat#555482, or cat#560976, clone HI30, lots# 9077614, 1285678, 7058663, 8232661, 20uL), CD34 PE (cat# 560941, clone 581, lots#7033536, 203612, 8221866, 20uL), CD19 PE (cat# 555413 or cat#561741clone HIB19, lots#8081579, 6124709, 6124705, 20uL), CD11b PE (cat# 561001, clone ICRF44, lots#6251566, 0066711, 20uL), HLA-PC5.5 (cat#560652, clone G46-6, lots# 7152686, 8159976, 30984, 1193612, 5uL).<br><br>TIMP2 ELISA kit Abcam cat# 100653 VEGF-A, VEGF-D, PlGF, TIE-2, IL-2, IL-4, IL-6, IL-8, IL-10, IL-12/p70, IL-13, IL-1?, TNF-?, and IF-? (V-PLEX and V-PLEX Plus Angiogenesis Panel 1 (Human) kits cat#15190G, 15190D, and V-PLEX Plus Proinflammatory Panel 1 Human Kit cat# K15049G-1 from Meso Scale Discovery (MSD), Maryland, USA),
Validation	All antibodies used in this study were commercially available, and have been stated to recognize the human analyte target for each intended assay application.1. CD105 PE, cat#560839, clone 266<br>Description: The 266 monoclonal antibody specifically binds to CD105. CD105 is a type I transmembrane glycoprotein that is encoded by END (Endoglin) and belongs to the transforming growth factor-β (TGF-β) type III receptor family. CD105 is expressed on cells as a homodimer comprised of ~95 kDa subunits. CD105 is expressed on vascular endothelial cells and placental syncytiotrophoblasts and

at lower levels on stromal fibroblasts. It is also expressed on mesenchymal stem cells, erythroid precursors, activated macrophages, pre-B cells, and some tumor cells and cell lines including U937 cells. CD105 serves as a regulatory component of the TGF-β receptor system. In association with TGF- βRI or TGF- βRII, CD105 binds TGF-β1 and TGF-β3 with high affinity but does not bind to TGF-β2. Expression of CD105 is increased on activated endothelium in tissues undergoing angiogenesis, such as in tumors, or in cases of wound healing or dermal inflammation.

Flow cytometric analysis of CD105 expression on
human U937 cells. U937 cells (ATCC, Cat No.
CRL-1593.2) were stained with PE Mouse anti-Human CD105 antibody (Cat. No. 5060839; solid line histogram) or a PE mIgG1, κ isotype control (Cat. No. 554680; dashed line histogram). Flow cytometric fluorescence histograms were derived from gated events with the forward and side light-scatter characteristics of viable cells. Flow cytometry was performed using a BD LSRTM II flow cytometry system.

2. CD90 FITC, cat#555595, clone 5E10
Description: The 5E10 monoclonal antibody specifically binds to human CD90 which is also known as Thy-1. CD90 is a 25-35 kDa glycophosphatidylinositol-anchored membrane glycoprotein of the Ig superfamily that is expressed on 1-4% of human fetal liver cells, cord blood cells, and bone marrow cells. The anti-CD90 antibody binds to a subset of immature CD34+ cells and a distinct subset of mature CD34- cells that are CD3+CD4+. The CD90+CD34+ population is highly enriched for cells capable of long-term culture. The anti-CD90 antibody is useful for enriching high proliferative potential colony-forming cells (HIPP-CFC) that are primative progenitor cells.

Flow cytometric analysis of CD90 expression on HEL
cell line. HEL cells were stained with either FITC Mouse IgG1, κ Isotype Control (Cat. No. 555748; dashed line histogram) or FITC Mouse Anti-Human CD90 (Cat. No. 555595/561969; solid line histogram). Fluorescent histograms were derived from gated events with the side and forward light-scatter characteristics of viable HEL cells.

or cat#328108, clone 5E10

Human Erythroleukemic cell line (HEL) stained with 5E10 FITC

3. CD73 APC, cat# 560847 Clone AD2
Description: The AD2 monoclonal antibody specifically binds to ecto-5'-nucleotidase, a 70 kDa, glycosyl phosphatidylinositol (GPI)-anchored glycoprotein. CD73 is expressed on subsets of T and B lymphocytes, follicular dendritic cells, epithelial cells, endothelial cells and mesenchymal stem cells. Its expression on lymphocytes increases during T and B cell development. CD73 has enzymatic activity and catalyzes the dephosphorylation of adenosine monophosphate (AMP) converting it to adenosine. It has been suggested that CD73 can mediate costimulatory signals in T cell activation and adhesion of lymphocytes to endothelium.

Flow cytometric analysis of CD73 on human lymphocytes. Whole blood was stained with APC Mouse anti-Human CD73 (Cat. No. 560847; solid line fluorescence histogram) and compared with whole blood stained with APC Mouse IgG1, κ Isotype Control (Cat. No. 554681; used at a matching concentration; dashed line histogram). The erythrocytes were lysed with BD PharmLyseTM Lysing Buffer (Cat. No. 555899). Flow cytometric fluorescence histograms were derived from gated events with the forward and side light-scatter characteristics of viable lymphocytes. Flow cytometry was performed using a BD LSRTM II flow cytometry system.

OR CD73 cat# 344002 Clone AD2

Human peripheral blood lymphocytes stained with AD2 PE and CD3 APC

Human peripheral blood lymphocytes stained with AD2 PE and CD19 APC

4. CD45 FITC, cat#555482, 560976 clone HI30
Description: The HI30 monoclonal antibody specifically binds to the 180, 190, 205, 220 kDa protein isoforms of CD45. CD45 is encoded by the PTPRC (Protein tyrosine phosphatase receptor type C) gene. CD45, also known as the leukocyte common antigen (LCA), is a member of the protein tyrosine phosphatase (PTP) family. It is present on all human leukocytes including lymphocytes, monocytes, granulocytes, eosinophils, and thymocytes. CD45 is absent from circulating erythrocytes, platelets, or mature erythroid cells of bone marrow and non-hemopoietic tissues.

Flow cytometric analysis of CD45 expression on human peripheral blood lymphocytes. Human whole blood was stained with either FITC Mouse IgG1, κ Isotype Control (Cat. No. 555748; dashed line histogram) or FITC Mouse Anti-Human CD45 antibody (Cat. No. 555482/560976/561865; solid line histogram). The erythrocytes were lysed with BD FACSTM Lysing Solution (Cat. No. 349202). The fluorescence histograms were derived from events with the forward and side light-scatter characteristics of intact lymphocytes.

5. CD34 PE, cat# 560941, clone 581
Description: The 581 monoclonal antibody specifically binds to CD34, a sialomucin-like type I transmembrane glycoprotein. This single-chain, 105-120 kDa, heavily O-glycosylated protein is expressed on hematopoietic progenitor cells, vascular endothelium, bone marrow stromal cells and embryonic fibroblasts. The cytoplasmic region of the CD34 antigen is a target for phosphorylation by activated protein kinase C suggesting CD34 may play a role in signal transduction. CD34 may also play a role as an adhesion molecule

since it binds to CD62E and CD62L. Clone 581 binds to the class III CD34 epitope. It is resistant to neuraminidase, chymopapain and glycoprotease. The 581 antibody blocks reactivity of another anti-CD34 monoclonal antibody, 8G12.

Flow cytometric analysis of CD34 expression by human peripheral blood mononuclear cells. Human PBMCs were stained PE Mouse Anti-Human CD34 antibody (Cat. No. 555822/560941). Flow cytometric dot plot showing side-scattered light versus CD34 were derived from gated events based on the light scattering characteristics viable cells. Flow cytometry was performed on a BD FACScanTM system.

11. CD19 PE, cat# 555413 or cat#561741, clone HIB19
Description: The HIB19 monoclonal antibody specificially binds to the 95 kDa type I transmembrane CD19 glycoprotein. CD19 is expressed during all stages of B-cell maturation and differentiation, except on plasma cells. CD19 is also present on follicular dendritic cells. It is not found on T cells or on normal granulocytes. CD19 is a signal transduction molecule that regulates B cell development, activation, proliferation and differentiation. It associates with the complement receptor 2 (CD21), TAPA-1 (CD81), Leu 13, and/or MHC class II to form a signal transduction complex on the surface of B cells. Anti-CD19 clone HIB19 partially blocks the binding of clone B43, another CD19-specific monoclonal antibody.

Profile of peripheral blood lymphocytes analyzed on a FACScan (BDIS, San Jose, CA)

12. CD11b PE, cat# 561001, clone ICRF44
Description: The ICRF44 monoclonal antibody specifically binds to CD11b, the 165-kDa adhesion glycoprotein that associates with the 95-kDa integrin β2 (CD18) to form the CD11b/CD18 complex, also known as Mac-1 or CR3. CD11b is a type I transmembrane glycoprotein that is encoded by ITGAM (Integrin alpha M). It is expressed on activated lymphocytes, monocytes, granulocytes, and a subset of NK cells. CD11b functions in cell-cell and cell-substrate interactions and is a receptor for iC3b, CD54 (ICAM-1), CD102 (ICAM-2) and CD50 (ICAM-3). This antibody significantly inhibits polymorphonuclear leukocyte aggregation in response to fMLP. This clone also cross-reacts with granulocytes, a subset of peripheral blood lymphocytes and some monocytes of baboon, and both rhesus and cynomolgus macaque monkeys. The distribution on lymphocytes and granulocytes is similar to that observed with peripheral blood from normal human donors. There are fewer CD11b-positive monocytes present in the non-human primate blood than in normal human donor samples.

Flow cytometric analysis of CD11b expression on human lymphocytes (Left Plot) or granulocytes (Right Plot). Human whole blood was stained with either PE Mouse IgG1, κ Isotype Control (Cat. No. 555749; dashed line histogram) or PE Mouse Anti-Human CD11b (Cat. No. 555388/561001/557321; bold line histogram). Erythrocytes were lysed with Pharm LyseTM Lysing Buffer (Cat. No. 555899). Fluorescence histograms depicting CD11b (or Ig isotype control) expression were derived from gated events with the side and forward light-scattering characteristics of viable lymphocytes or granulocytes. Flow cytometry was performed on a BD FACScanTM system.

13. HLA-PC5.5 (cat#560652, clone G46-6
The G46-6 monoclonal antibody specifically binds to HLA-DR, a major histocompatibility complex (MHC) class II antigen. HLA-DR antigens are encoded by genes within the Human Leukocyte Antigen (HLA) Complex located on chromosome 6. HLA-DR is a transmembrane heterodimeric glycoprotein composed of an α chain (36 kDa) and a β subunit (27 kDa) expressed primarily on antigen presenting cells: B cells, dendritic cells, monocytes, macrophages, and thymic epithelial cells. HLA-DR is also expressed on activated T cells. This molecule plays a major role in mediating cellular interactions during antigen presentation to CD4-positive T cells.

Flow cytometric analysis of HLA-DR on human lysed whole blood. Human whole blood was lysed with BD FACSTM Lysing Solution (Cat. No. 349202) and stained with the PerCP-CyTM5.5 Mouse Anti-Human HLA-DR antibody (Cat. No. 560652/552764; unshaded histogram) or with a PerCP-CyTM5.5 Mouse IgG2a, κ isotype control (Cat. No. 550927; shaded histogram). Fluorescent histograms showing expression of HLA-DR (or Ig isotype staining) were derived from gated events based on forward and side light scattering characteristics for intact lymphocytes. Flow cytometry was performed on a BDTM LSR II flow cytometry system.

Eukaryotic cell lines

Policy information about cell lines and Sex and Gender in Research

Cell line source(s)

Lomecel-B is an investigational allogeneic adult human mesenchymal stem cell (hMSC) therapy, comprised of a suspension of human bone marrow-derived mesenchymal stem cells27,29,48,58. Lomecel-B is obtained from bone marrow aspirates from the iliac crest of unrelated and human leukocyte antigen (HLA)-unmatched healthy adult donors (ages 18-45). Marrow donor

characteristics and cell count are provided in Table S13. All bone marrow collections are regulated under 21 CFR 1271, "Human Cells, Tissues, and Cellular and Tissue Based Products (HCT/Ps). All donors are screened in accordance with 21 CFR 1271 Subpart C Donor Eligibility. The material is shipped to Longeveron for processing under aseptic conditions for further processing in a GMP facility. Lomecel-B MSCs were isolated from bone marrow using density gradient separation. The sex of each donor cell line was: LMSC024 (F) LMSC037 (M) LMSC040 (F) LMSC042 (M) LMSC045 (M) LMSC053 (M) LMSC055 (M) LMSC064 (F). See Table S13 and Materials and methods.

Authentication

Lomecel-B is culture expanded in complete medium (alpha-minimum essential medium (MEM; Gibco), containing FBS (fetal bovine serum; Corning)) and grown to approximately 85-90% confluency in multilayer vessels. At each passage, Lomecel-B is trypsinized, pooled, subjected to cell count and viability assessments, and multilayer vessels are then seeded. All passages utilize the same complete medium. All passages must have cell viability ≥70%. At harvest, conditioned media (supernatant) samples are collected and frozen at -80°C, then Lomecel-B is trypsinized, pooled, subjected to cell count/viability assessment and filled at specific concentrations into CS50 cryobags in a 20mL suspension containing a cryoprotectant composed of Hespan, HSA, and DMSO. Lomecel-B is frozen utilizing-controlled rate freezers and stored at ≤ -135°C in vapor phase LN2 freezers. Each lot of Lomecel-B is tested to confirm MSC (mesenchymal stem cell; also known as medicinal stem cell) identity and ensure all critical quality attributes meet product specifications (Table S13). If all specifications are met the cryopreserved product is released. Identity is performed via immunophenotyping via flow cytometry. Lomecel-B lots must be positive for surface cell markers, CD105, CD90, and CD73 (≥95%). Lomecel-B must be negative (≤2%) for CD45, CD11b, CD19, Human Leukocyte Antigen DR Isotype HLA-DR (MHC II cell surface receptor) and (≤5%) for CD34. Lomecel-B must be negative for mycoplasma, adventitious viruses via culture on three cell types and negative for Parvo B19, HIV 1, HIV 2, Hepatitis B, Hepatitis C, HTLV 1, HTLV 2, CMV and EBV by PCR, pass USP 71 sterility with no growth and have post thaw viability ≥ 70%.

Mycoplasma contamination

All cell lines tested negative for mycoplasma (see Table S13).

Commonly misidentified lines (See ICLAC register)

n/a

Clinical data

Policy information about clinical studies
All manuscripts should comply with the ICMJE guidelines for publication of clinical research and a completed CONSORT checklist must be included with all submissions.

Clinical trial registration

NCT05233774

Study protocol

The study protocol can be found in the trial master file at ClinicalTrials.gov

Data collection

Patient enrollment occurred at 10 clinical centers in South Florida from Martin County to Miami-Dade County between December 29, 2021, and November 21, 2022, with final follow-up on August 21, 2023.

Outcomes

The primary endpoint entails tabulation of the percentage of patients having SAEs within 4 weeks after any infusion with investigational product (Lomecel-B or placebo) by treatment group. Rate is calculated on subject-level (number and percentage of patients experiencing an SAE within 4 weeks of any infusion). Rates and Clopper Pearson exact confidence intervals for each treatment group will be analyzed.

The secondary endpoint is the composite AD score (CADS) for dementia progression at 39 weeks, and was calculated by combining z-scores for change from baseline (to study endpoint) values for multiple assessments: ADCS-ADL, CDR-SB, ADAS-Cog-13, and left hippocampal volume. Each component comprised 25% of the final score (i.e., each equally weighted). Inverted measures of the ADAS-Cog-13 and CDR-SB will be used in order to match directionality of the other measures with respect to improvement/decline changes.

Flow Cytometry

Plots

Confirm that:

☒ The axis labels state the marker and fluorochrome used (e.g. CD4-FITC).

☒ The axis scales are clearly visible. Include numbers along axes only for bottom left plot of group (a 'group' is an analysis of identical markers).

☒ All plots are contour plots with outliers or pseudocolor plots.

☒ A numerical value for number of cells or percentage (with statistics) is provided.

Methodology

Sample preparation

Cells were thawed using thaw media; cell counts and viability were analyzed using a cell counter (NucleoCounter; NC-200; ChemoMetec, Allerod, Denmark). Cells were washed with flow staining buffer (cat # 00422226, Thermo Fisher) not to exceed 50 mL and centrifuged at 500 x g for 10 min at 20°C. After centrifugation, cell pellets were resuspended in flow buffer at a concentration of 1x106 to 1x107 cells per mL. 100uL of cells were added to each tube with appropriate antibodies and incubated for 60 min. in the dark at 2-4°C. Cells were labeled with live/dead dye; 7-AAD (cat# 559925); negative controls included: FITC IgG1 (cat#556649), PE IgG1 (cat#556650), PCP5.5 IgG1 (cat#552834), APC IgG1 (cat#550854). The live/dead and all isotype control IgG1 antibodies were used undiluted at 20uL. The cells were labeled with cell surface marker

antibodies for CD105 PE (cat#560839, 5uL), CD90 FITC (cat#555595, 5uL), CD73 APC (cat# 560847, 5uL), CD45 FITC (cat#555482, 20uL), CD34 PE (cat# 560941, 20uL), CD19 PE (cat# 555413, 20uL), CD11b PE (cat# 561001, 20uL), HLA-PC5.5 (cat#560652, 5uL). Unstained cells served as an additional experimental control. All cell surface marker antibodies were used undiluted and were purchased from BD Biosciences. After the incubation step, cells were washed with 1 mL of flow cytometry staining buffer solution and centrifuged for 10 min at 500 x g, at room temperature. Cell pellets were resuspended in 300uL flow cytometry staining buffer. All samples were run in triplicates.

Instrument	CytoFLEX V5-B5-R3
Software	CytExpert and FlowJo
Cell population abundance	Similar to the definition of bone marrow derived MSCs following the ISCT criteria, passing Lomecel-B lots must be positive for the surface cell markers CD105, CD90, and CD73 (≥95%). Lomecel-B must be negative (≤2%) for CD45, CD11b, CD19, and ≤5% for CD34.
Gating strategy	A standard gating strategy was employed using side scatter (SSC)-H and -A and forward scatter (FSC)-H, where cells were first subcategorized as single cells vs doublets, further categorized as live cells using a live/dead dye, and assessed as positive using a gate permitting a maximum of 0.01% of unstained cells.

☒ Tick this box to confirm that a figure exemplifying the gating strategy is provided in the Supplementary Information.

Magnetic resonance imaging

Experimental design

Design type	Resting state
Design specifications	This was a longitudinal vMRI and DTI assessment at baseline, 16, 26, and 39 weeks after initiation of the treatment regimens, with vMRI and DTI data collected at each timepoint.
Behavioral performance measures	n/a: No behavioral tasks were provided to study participants during MRI imaging.

Acquisition

Imaging type(s)	Structural (volumetric), and diffusion tensor imaging
Field strength	3T
Sequence & imaging parameters	3DT1 MPRAGE : Sagittal acquisition with 176 slices, FOV = 240mmx240mm, Slice thickness = 1.2mm, TR/TE = 1800ms/2.49ms, TI = 900ms, flip angle = 10 degrees, matrix size = 192x192
Area of acquisition	Whole brain vMRI scans were used in this study, with atlas-based sub-region identification. Volumetric MRI and Atrophy analysis: All 3DT1 scans were N4 bias corrected, registered to a 256mm^3 template space and isotropically resampled to achieve a voxel size of 1mm^3. Preprocessed 3DT1 scans were then analyzed in FreeSurfer 6 to generate baseline regional volume measures of all available cortical/sub-cortical, white matter, and ventricular regions. For atrophy measures, follow up imaging timpoints are registered with baseline scans to a log-symmetric midway space for tensor based morphometry analysis. All volume and atrophy measures are reported in mm^3 and converted to cm^3 as applicable.

Diffusion MRI ☒ Used ☐ Not used

Parameters

DTI Parameters: Axial spin echo, echo planar imaging acquisition with 80 slices, FOV = 232mmx232mm, Slice thickness = 2mm, Phase encode direction = AP, TR/TE = min/min (but still T2 weighted, dependent on scanner performance), matrix size = 116x116, bandwidth = 1486 Hz/Px, directions = 30, b-value = 1000 s/mm^2, 2 b0 averages. In addition, a DTI b0 reverse sequence with identical parameters (including TR/TE/BW)but with phase encode direction = PA.

DTI analysis:

A standardized DTI sequence (2mm^3 voxel size, 30 directions, b-value =1000 s/mm^2) was deployed to each site in conjunction with a reverse phase encode DTI sequence (blip down) with identical contrast parameters (TR/TE/Bandwidth) for distortion correction. Scans were received and quality controlled to ensure adherence to approved acquisition settings. Pre-processing included eddy and motion correction that was performed using a linear affine transformation on all gradient images with the baseline image of b=0 s/mm^2 (1). The reverse encoding DTI image (blip down acquisition) was employed for correcting the distortions in the main DTI image (blip up acquisition) using DRBUDDI which is based on symmetric diffeomorphic registration (1-3) to estimate the distortion field. Diffusion tensors were then computed using a standard least square approximation and the scalar maps of fractional anisotropy (FA) , mean diffusivity (MD) axial diffusivity (AD) and radial diffusivity (RD) were derived from the tensor image. The FA maps were then registered to the Freesurfer (4) processed 3DT1 image in standard 256*256*256 space and the deformation was applied to remaining maps (MD, AD and RD). Finally, the FA image was registered to JHU atlas's (6) FA image using ANTS (5) SyN deformable registration and the deformation was applied to all the maps. This allowed for a seamless overlay of cortical, subcortical, and white matter labels for regional value extraction. For each subject, the registrations and overlays were checked manually, and reprocessed or rejected in case of failures.

1. C. Pierpaoli, L. Walker, M. O. Irfanoglu, A. Barnett, P. Basser, L-C. Chang, C. Koay, S. Pajevic, G. Rohde, J. Sarlls, and M. Wu,

2010, TORTOISE: an integrated software package for processing of diffusion MRI data, ISMRM 18th annual meeting, Stockholm, Sweden, abstract #1597, (pdf)

2. Mustafa Okan Irfanoglu1,2,3, Amritha Nayak1,2,3, Jeffrey Jenkins1,2, and Carlo Pierpaoli1,2 ,TORTOISEv3:Improvements and New Features of the NIH Diffusion MRI Processing Pipeline, ISMRM 25th annual meeting, Honolulu, HI, abstract #3540,(pdf)

3. Irfanoglu MO, Modi P, Nayak A, Hutchinson EB, Sarlls J, Pierpaoli C.DR-BUDDI (Diffeomorphic Registration for Blip-Up blip-Down Diffusion Imaging) method for correcting echo planar imaging distortions, Neuroimage. 2015 Feb 1;106:284-99. doi: 10.1016/j.neuroimage.2014.11.042. Epub 2014 Nov 26. (pdf)

4. Fischl, Bruce. "FreeSurfer." Neuroimage 62.2 (2012): 774-781.

5. Avants, Brian B., Nick Tustison, and Gang Song. "Advanced normalization tools (ANTS)." Insight j 2.365 (2009): 1-35.

6. Mori, Susumu, et al. "Stereotaxic white matter atlas based on diffusion tensor imaging in an ICBM template." Neuroimage 40.2 (2008): 570-582.

Preprocessing

Preprocessing software

Detailed description provided above. For the 3DT1 scans, FreeSurfer 6 was used to generate the segmentation maps. Tensor based morphometry measures were calculated using in-house created software. DTI analysis leveraged DRBUDDI for distortion correction and ANTS for registration. DTI calculations were derived using in-house created software.

Normalization

3DT1 scans were normalized to MNI average_305 template per FreeSurfer's processing steps. The JHU atlas was used for registration within the DTI pipeline to allow for cortical/sub-cortical gray matter statistics in addition to the white matter tracts defined in JHU space.

Normalization template

Described above but the MNI 305_template and JHU templates were used.

Noise and artifact removal

Based on the definition here, no noise/artifact removal was performed. All calculated maps were visually QCed for accuracy of the derived result.

Volume censoring

Not used

Statistical modeling & inference

Model type and settings

MMRM. The model included fixed effects for visit, treatment group (4 level variable), visit by treatment interaction, sex, and baseline value of the outcome parameter. Patient was included as a random effect.

Effect(s) tested

Least-squares (LS) means, standard errors (SE), and 95% CIs for mean change from baseline were obtained from the model for each treatment group at each visit, including the pooled Lomecel-B treatment group. The LS-mean differences, SE, 95% CIs, and two-sided p-values for the differences between treatment groups were obtained for each active treatment group (Group 2, 3 and 4) relative to placebo (Group 1) at each visit. The pooled Lomecel-B treatment group effect relative to placebo was obtained from the same model.

Specify type of analysis: ☐ Whole brain ☒ ROI-based ☐ Both

Anatomical location(s)	For volumetric MRI the following regions were calculated (left, right, and bilateral):<br>• Whole Brain<br>• Lateral Ventricles<br>• Hippocampus<br>• Whole Gray Matter<br>• Cingulate cortex: Caudal anterior cingulate, Rostral anterior cingulate, Isthmus cingulate, Posterior cingulate<br>• Frontal cortex: Superior frontal, Caudal middle frontal, Rostral middle frontal, Pars opercularis, Pars orbitalis, Pars triangularis, Lateral orbitofrontal, Medial orbitofrontal, Frontal pole, Precentral gyrus, Paracentral lobule<br>• Medial temporal cortex: Hippocampus, Amygdala, Entorhinal, Parahippocampal gyrus, Temporal pole, Fusiform<br>• Temporal cortex: Entorhinal, Parahippocampal gyrus, Temporal pole, Fusiform, Inferior temporal, Middle temporal, Superior temporal, Transverse temporal, Banks of the superior temporal sulcus<br>• Occipital cortex: Lateral occipital, Lingual gyrus, Cuneus, Pericalcarine sulcus<br>• Parietal cortex: Inferior parietal, Superior parietal, Postcentral gyrus, Supramarginal gyrus, Precuneus<br>• Striatum: Caudate, Putamen<br>• Thalamus<br>For DTI all available FreeSurfer regions were reported for FA,MD,AD,and RD in addition to free water fraction and free water corrected values. For JHU the following white matter tracts were calculated (left,right,bilateral where appropriate):<br>• Whole White Matter<br>• Cerebral Peduncle<br>• Cingulum<br>• Corona Radiata (Anterior, Posterior, Superior, Whole)<br>• Corpus Collosum (Body, Genu, Splenium, Whole)<br>• Corticospinal tract<br>• External Capsule<br>• Fornix<br>• Inferior Cerebellar Peduncle<br>• Internal Capsule (Anterior, Posterior, Retrolenticular, Whole)<br>• Medial Lemniscus<br>• Middle Cerebellar Peduncle<br>• Pontine Crossing Tract<br>• Sagittal Stratum<br>• Superior Cerebellar Peduncle<br>• Superior Fronto-Occipital Fasciculus<br>• Superior Longitudinal Fasciculus<br>• Tapetum<br>• Thalamic Radiation (Posterior)<br>• Uncinate Fasciculus
Statistic type for inference<br>(See Eklund et al. 2016)	Voxel-wise
Correction	None used.

Models & analysis

n/a	Involved in the study
☐	☒ Functional and/or effective connectivity
☒	☐ Graph analysis
☐	☒ Multivariate modeling or predictive analysis

Functional and/or effective connectivity	To determine whether reduced brain atrophy correlated with improved clinical scores, we performed a Pearson correlation analysis.
Multivariate modeling and predictive analysis	Change from baseline in CADS was statistically analyzed with a mixed model for repeated measures (MMRM) analysis. The model included fixed effects for visit, treatment group (4 level variable), visit by treatment interaction, sex, and baseline value of the outcome parameter. Patient was included as a random effect. Similar MMRM analyses were conducted for the exploratory efficacy endpoints of ADAS-cog-13, MMSE-2, ADCS-ADL, CDR-SB, MoCA, NPI, QOL-AD (both caregiver and study subject), ADCS-ADL, ADRQL, brain volumetry (via MRI), diffusion tensor imaging.
